# Distinct mural cells and fibroblasts drive fibrochondrogenesis in retrodiscal tissue following temporomandibular joint disc displacement

**DOI:** 10.1172/jci.insight.196343

**Published:** 2026-02-10

**Authors:** Wenlin Yuan, Yilin Chen, Ruojin Yan, Wei Liu, Chenyu Wang, Ying Wang, Qiaoli Dai, Wen Li, Mengqi Zhu, Xiao Chen, Jiejun Shi

**Affiliations:** 1 Stomatology Hospital, School of Stomatology, Zhejiang University School of Medicine, Zhejiang Provincial Clinical Research Center for Oral Diseases, Key Laboratory of Oral Biomedical Research of Zhejiang Province, Cancer Center of Zhejiang University, Engineering Research Center of Oral Biomaterials and Devices of Zhejiang Province, Hangzhou, China.; 2 Dr. Li Dak Sum & Yip Yio Chin Center for Stem Cells and Regenerative Medicine and Department of Orthopedic Surgery of Sir Run Shaw Hospital, Zhejiang University School of Medicine, Hangzhou, China.; 3 Liangzhu Laboratory, Zhejiang University, Hangzhou, China.; 4 Key Laboratory of Tissue Engineering and Regenerative Medicine of Zhejiang Province, and; 5 Department of Sports Medicine, Zhejiang University School of Medicine, Hangzhou, China.; 6 China Orthopedic Regenerative Medicine Group (CORMed), Hangzhou, China.; 7 Department of Sports Medicine & Orthopedic Surgery, The Second Affiliated Hospital, and Dr. Li Dak Sum & Yip Yio Chin Center for Stem Cells and Regenerative Medicine, Zhejiang University School of Medicine, Hangzhou, China.

**Keywords:** Bone biology, Cell biology, Cartilage, Extracellular matrix

## Abstract

Adaptive remodeling of retrodiscal tissue following anterior disc displacement (ADD) of the temporomandibular joint (TMJ) has been recognized for decades, yet the underlying cellular dynamics and molecular mechanisms remain unclear. Using a porcine ADD model, this study investigated the cellular and molecular basis driving retrodiscal tissue adaptation. Histological staining revealed adaptive remodeling of retrodiscal tissue after ADD induction, with dense connective tissue and cartilaginous masses replacing loose connective tissue. Single-cell RNA-Seq captured pronounced fibroblast expansion during tissue remodeling, notably the FB2 subcluster with high developmental potential, and the emergence of a mural cell subcluster, MC4, associated with extracellular matrix (ECM) remodeling. CellChat analysis highlighted MC4-FB2 crosstalk via FGF2 and BMP5 signaling. The combination of pathway-aware multilayered hierarchical network (P-NET) and Seurat with drug database screening identified 5 promising compounds. Among them, zaprinast demonstrated the most robust effects by enhancing the remodeling capability of fibroblasts in vitro and alleviated TMJ deformation in vivo. Collectively, fibroblast activation is pivotal for early retrodiscal tissue adaptation after ADD, which is driven by MC4-derived FGF2/BMP5 signaling. Zaprinast treatment potentiates this remodeling process. These findings provide potentially new insights into the cellular basis of TMJ adaptation and identify potential therapeutic targets for ADD management.

## Abstract 2

Fibroblast activation is pivotal for retrodiscal tissue adaptation following temporomandibular joint disc displacement, mediated by pro-fibrotic/chondrogenic mural cell-derived FGF2/BMP5 signaling, while Zaprinast potentiates this process.

## Graphical abstract

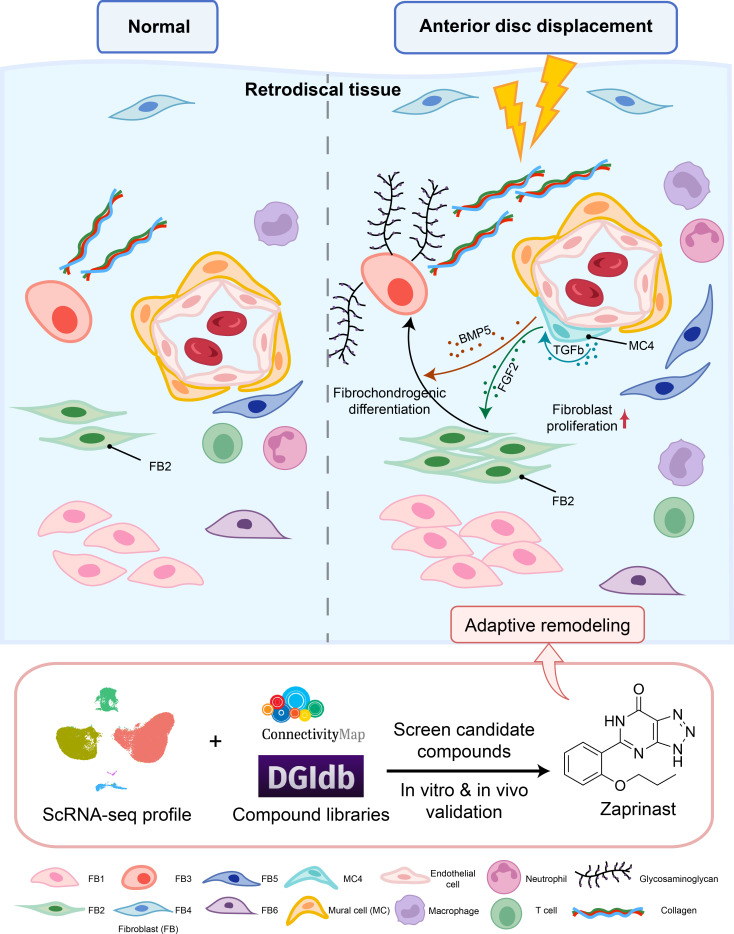

## Introduction

Anterior disc displacement (ADD) of the temporomandibular joint (TMJ) is the most common subtype of temporomandibular disorder, characterized by the forward shifting of the disc from its normal position (1, 2). Studies have reported the association between ADD and the development of osteoarthritis (3). MRI findings in patients with ADD revealed joint effusion and degenerative changes including bone erosion, grinding, and osteophyte formation (4). However, clinical observations indicate that some patients with ADD remain asymptomatic or only exhibit transient symptoms, with many having long-term TMJ stability (5). Variations in clinical outcomes suggest that individuals have differential adaptive capacities in response to traumatic intraarticular movements, which are closely related to the response of the retrodiscal tissue (RT).

The RT is attached posterior to the articular disc. It is mainly composed of loose connective tissue, richly vascularized, and covered by synovial tissue (6). Compared with the disc, its load-bearing capacity is much weaker (7). After ADD, part of the RT is positioned above the condyle and subjected to abnormal stress. Under this condition, the RT may undergo two distinct types of changes. One is destructive, characterized by degenerative changes involving the breakdown of collagen or elastic fibers, often accompanied by inflammation. The RT eventually becomes thinner or even perforated, resulting in persistent pain and dysfunction (8). The other is adaptive, including conversion of the loose connective tissue to dense connective tissue or even forming a fibrocartilage-like structure capable of bearing abnormal loads (9, 10). When tissue remodeling is sufficient, the RT may acquire structural similarities to the disc, thereby expanding the range of area that can withstand stress during condyle motion. Thus, some researchers have proposed that enhancing adaptive remodeling of RT by eliminating unfavorable factors can achieve a therapeutic effect even if the disc displacement is not resolved.

Adaptive remodeling of the RT has been recognized for decades. In a rat ADD model, the RT progressively transformed into dense fibrous tissue with marked reduction in adipose content 8 weeks after ADD induction (9). Gu et al. (10) observed chondrocyte differentiation in the RT by detecting type II collagen mRNA expression in a rabbit ADD model at 1 week, and typical cartilage tissue was apparent by 2 weeks. A longitudinal study of patients with ADD without reduction over a period of 4–8 years revealed pseudo-disc–like changes in the RT in 45% of patients (11). Histological examinations have also documented the presence of dense fibrous tissue containing scattered chondrocytes and increased glycosaminoglycan (GAG) content in some patients with ADD (12, 13). Nevertheless, the cellular and molecular mechanisms driving this remodeling process remain poorly understood.

Compared with rodents, porcine TMJ more closely resembles human TMJ in bilateral occlusion, translational movement, disc structure, and biomechanical function (14, 15). These characteristics make pigs particularly suitable for temporomandibular disorder research. Moreover, suture-induced ADD animal modeling fails to replicate human disease progression, as the traction forces generated are inconsistent and difficult to control. In contrast, elastic traction allows quantifiable force adjustment and provides sustained force to produce stable and reproducible results (16). In this study, we established a porcine unilateral ADD (UADD) model using surgical intervention with a titanium spring, and performed single-cell RNA-Seq (scRNA-Seq) analysis of TMJ disc and RT 5 weeks after ADD induction. We aimed to resolve the cellular heterogeneity in disc and RT and capture critical transcriptional changes and cellular drivers promoting adaptive remodeling of RT.

## Results

### ScRNA-Seq resolves cell heterogeneity in porcine discs and RTs.

**Figure 1:**
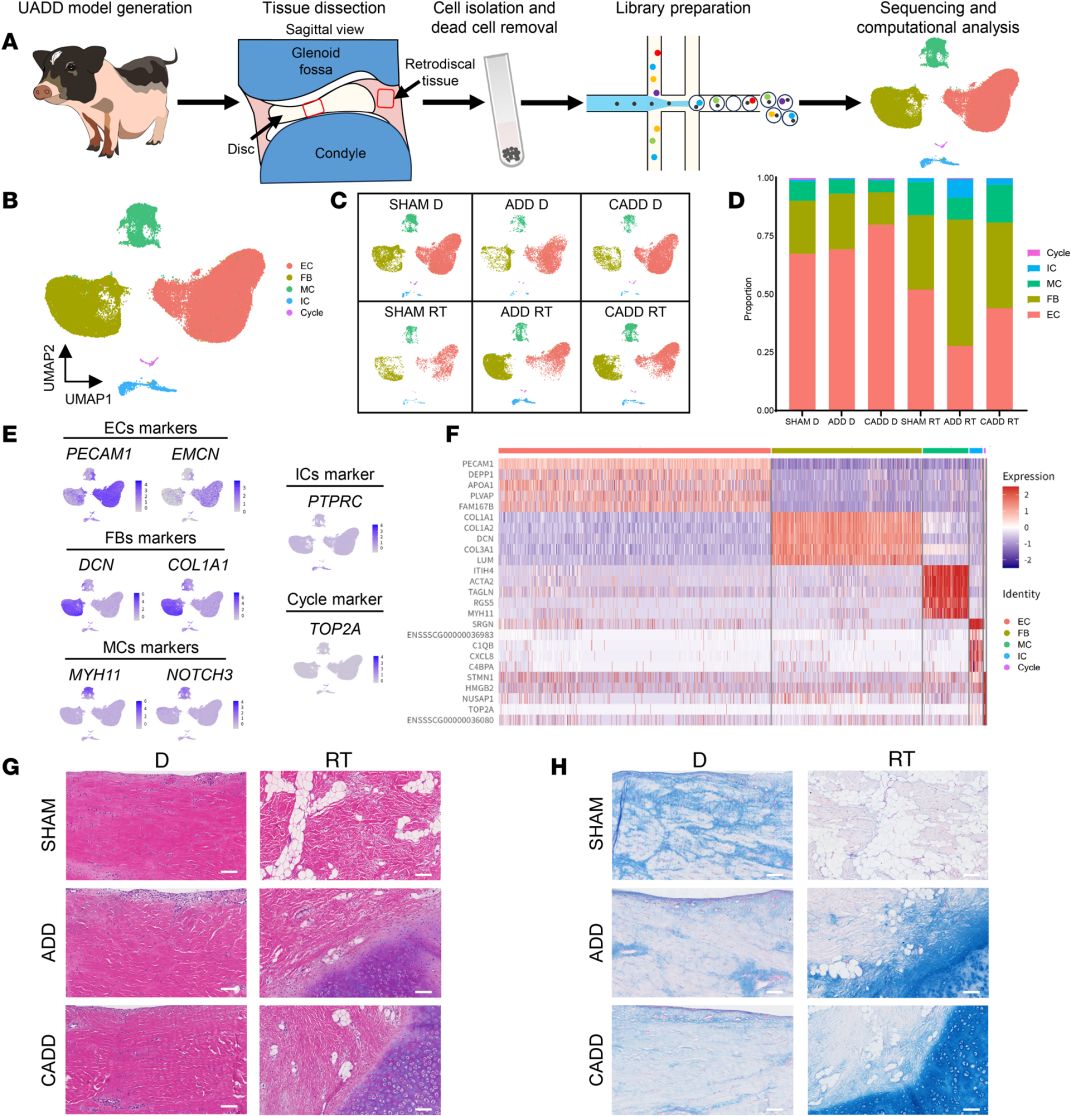
The cellular landscape and histological features of porcine TMJ discs and RTs from the sham, ADD, and CADD groups. (**A**) A schematic workflow illustrating the generation of the UADD pig model and subsequent dissection of pig TMJ discs and RTs for single-cell transcriptomic analysis. (**B**) Dimension reduction presentation of combined single-cell transcriptome data from TMJ discs and RTs of all groups. Each dot represents a single-cell and is labeled with corresponding cell categories and colored according to its cell-type identity. Five cell clusters are visualized by a uniform manifold approximation and projection (UMAP) plot: endothelial cells (ECs); fibroblasts (FBs); immune cells (ICs); mural cells (MCs); cells in cell cycle (Cycle). (**C**) Dimension reduction presentation of single-cell transcriptome data of TMJ discs (Ds) and retrodiscal tissues (RTs) from sham, ADD, and CADD groups via UMAP, displayed separately by tissue origins and groups. (**D**) Bar graph shows the fraction of cell clusters by tissue origins and groups. (**E**) Expression patterns of selected markers projected on the UMAP plot. (**F**) Heatmap revealing the top 5 differentially expressed genes (DEGs) of each cell cluster. Representative H&E staining (**G**) and Alcian blue staining (**H**) of the TMJ discs and RTs from the sham, ADD, and CADD groups. Scale bars: 100 μm.

To understand the composition and molecular profiles of cells in different regions and pathological states, we profiled cells from the sham operation and UADD models using scRNA-Seq. TMJ samples with sham operation were collected as the sham group. In the UADD model, TMJ samples from the ADD induction side (ADD group) and the contralateral side (CADD group) were collected. The overall workflow of the sample dissociation and scRNA-Seq is shown in Figure 1A. An unsupervised clustering algorithm partitioned the cells into 7 separated clusters (Supplemental Figure 1C; supplemental material available online with this article; https://doi.org/10.1172/jci.insight.196343DS1), which were classified into 5 principal cell types: endothelial cells (ECs), fibroblasts (FBs), mural cells (MCs), immune cells (ICs), and cells in the cell cycle (Figure 1, B and C). Markers used to annotate each cell cluster are presented in Figure 1E (17, 18). The top 5 differentially expressed genes (DEGs) in each cluster are shown in Figure 1F.

The distribution and proportion of cell clusters according to their origin were also evaluated. Stacked bar plots show the proportion of the main cell types in disc and RT (Figure 1D). Among major cell types, FBs and ECs comprised the vast majority of cells. Unlike rodents, whose TMJ discs are mostly composed of FBs, porcine discs contain a substantial vascular component. This is consistent with findings of recent studies that the TMJ disc of higher-order species is in fact vascularized and exhibits higher angiogenic gene expression compared with knee meniscus (14, 17).

### Adaptive remodeling occurs in RTs after ADD induction.

To define histological changes after ADD induction, we applied H&E staining and Alcian blue staining. The disc from the sham group was characterized by anterior-posterior fiber alignment in the intermediate zone, with spindle-shaped FBs and round-shaped chondrocyte-like cells distributed in the dense connective tissue. However, the RT from the sham group exhibited relatively loose and irregular connective tissue, with interspersed adipose tissue (Figure 1G). Furthermore, Alcian blue staining was observed in discs, with the intermediate zone showing the strongest intensity. In contrast, the RT had almost no Alcian blue staining (Figure 1H). Different GAG contents in disc and RT closely correlate with the mechanical demands they are subjected to (19). After ADD induction, small fissures were found among collagen fibers, with slightly reduced GAG content in the disc from ADD and CADD groups. These indicated weaken mechanical properties of the TMJ disc in both the ADD and contralateral sides. In RT from the ADD and CADD groups, parts of the loose connective tissue and adipose tissue were replaced by dense connective tissue and accompanied by FB proliferation. Concurrently, we found cartilaginous masses emerged in the RT by Alcian blue staining (Figure 1, G and H). Importantly, the condyle underneath did not exhibit overt degenerative changes after ADD induction (Supplemental Figure 2). Overall, these results revealed adaptive remodeling of the RT on both ADD and CADD sides, with fibrosis and chondrogenesis as the main manifestations.

We further investigated transcriptional changes in the disc and RT after ADD induction and captured changes in the cell proportion of the main cell types. Compared with the sham group, discs from the ADD and CADD groups showed minor changes in the main cell proportions, whereas RTs revealed remarkable changes, typically characterized by an increase in FBs (Figure 1D). These findings suggest that the RT is highly adaptive and sensitive to mechanical stress. Fibroblast proliferation in the RT likely facilitates load bearing during joint movement.

### Identification of responsive FB subtypes participating in adaptive remodeling of the RT.

**Figure 2:**
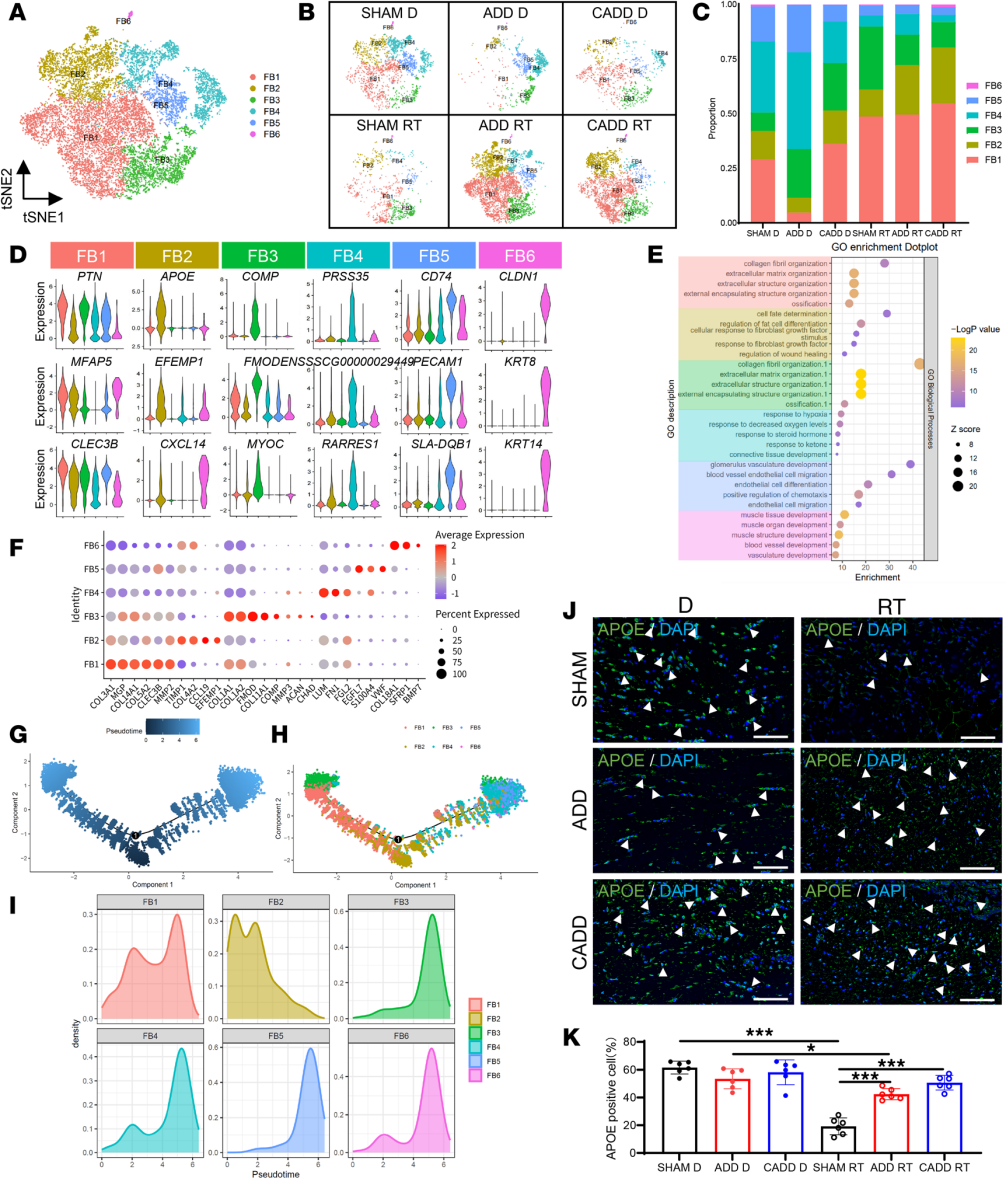
Characterization of FB subtypes participating in adaptive remodeling of the RT. (**A**) Dimension reduction presentation of combined single-cell transcriptome data of FB cluster from TMJ discs and RTs of all groups. Six subclusters of FBs are identified and visualized by a t-distributed stochastic neighbor embedding (t-SNE) plot. (**B**) Dimension reduction presentation of single-cell transcriptome data of FB cluster in sham, ADD, and CADD groups from TMJ discs and RTs via t-SNE, displayed separately by tissue origins and groups. (**C**) Bar graph shows the fraction of FB subclusters by tissue origins and groups. (**D**) Violin plots show the expression of differentially expressed genes (DEGs) in different FB subclusters. Expression values are normalized. (**E**) Dot plot of Gene Ontology (GO) Biological Process enrichment analysis of DEGs in different FB subclusters. (**F**) Expression of extracellular matrix (ECM) markers in different FB clusters. Monocle analysis shows the trajectory order of FBs colored by pseudo-time value (**G**) and cell type (**H**). (**I**) Density distribution of FB subclusters along pseudo-time. (**J**) Representative images of TMJ discs and RTs subjected to immunofluorescence staining for APOE in different groups and quantification (**K**); 3 random fields were selected from each of the 2 pig samples for measurement. Statistical significance was determined by 1-way ANOVA. Data represent mean ± SD. *P < 0.05, ***P < 0.001. Green, APOE; blue, DAPI; white arrowhead, cells expressing APOE. Scale bars: 100 μm.

Given that FBs are known to play central roles in ECM homeostasis and remodeling (20), we further investigated their composition in detail. Unsupervised clustering divided FBs into 6 subclusters (FB1–FB6) (Figure 2, A–C). The DEGs (Figure 2D) and Gene Ontology (GO) enrichment analysis (Figure 2E) of each FB subcluster were utilized to define their identities. FB1 represented the predominant FB population in both disc and RT, but its proportion in the disc decreased markedly after ADD. FB1 preferentially expressed *PTN*, supporting FB proliferation and function (21). FB2 was distinguished by signature genes related to cholesterol metabolism (*APOE*) and ECM integrity (*EFEMP1*) (22, 23). GO analysis further revealed that FB2 was enriched in functions related to cell differentiation, wound healing, and response to FB growth factor. In the sham group, FB2 proportions were similar in both disc and RT. In contrast, FB2 showed a substantial increase in the RT of ADD and CADD groups, indicating it is highly responsive and may play an active role in tissue remodeling. FB3 expressed *COMP* and *FMOD*, genes involved in collagen biosynthesis, ECM assembly, and cartilage composition (24, 25). FB4 displayed high expression of *PRSS35* and *ENSSSCG00000029449* (proteoglycan 4, *PRG4*), suggesting a role in cellular homeostasis under stress environment and lubrication (26, 27). Genes such as *CD74* and *PECAM1* were expressed by FB5, indicating its function in regulating cell migration and angiogenesis. FB6 accounted for a minor fraction of FBs and was associated with epithelial junctions and muscle development (Figure 2, D and E).

To better understand the function of porcine TMJ FBs, we compared their transcriptomic profiles with murine TMJ disc FBs at different postnatal stages (17). Correlation analysis demonstrated that porcine FB1 and FB2 were most similar to murine FB1 (FBs associated with fibrillogenesis and fibrillary homeostasis), whereas porcine FB3 resembled murine FB6 (chondrogenesis-related FBs). However, correlation coefficients were generally low (approximately 0.2), highlighting substantial interspecies heterogeneity (Supplemental Figure 3).

TMJ tissues undergo continuous ECM remodeling processes to maintain microenvironment homeostasis. Given the central role of FBs in ECM organization, we investigated ECM expression patterns across FB subtypes. The dot plot shows dynamic ECM gene expression among FB clusters (Figure 2F). The main constitutive collagen genes *COL1A1* and *COL1A2* were highly expressed in FB3 and moderately expressed in FB1. A small proportion of FB3 also expressed fibrocartilage-associated genes *ACAN*, *CHAD,* and *COMP*, suggesting FB3 as a key subcluster contributing to ECM composition. In contrast, the remaining FB subclusters primarily modulated ECM formation and assembly. FB2 expressed non-fibrillar collagen gene *COL4A2*, which functions in connective tissue anchoring and basal lamina formation (28). FB4 regulated collagen fibrillogenesis and ECM assembly by *LUM* and *FN1* expression (29). Meanwhile, FB5 and FB6 were enriched for secreted factors including *EGFL7*, *S100A4,* *SFRP1*, and *BMP7*, supporting roles in angiogenesis and myotendinous junction development (30, 31).

We further used pseudo-time trajectory analysis to predict differentiation states of FB clusters. FB2 has optimal development potential (Supplemental Figure 4), and FB2s serve as progenitor cells that differentiate in 2 directions, ECM-producing FBs (FB1, FB3) and modulatory FBs (FB4–6) (Figure 2, G–I). Studies have shown that EGF-like fibulin ECM protein 1 (EFEMP1) plays a role in maintaining the immature status of cells in the cartilage superficial zone, and apolipoprotein E (APOE) is a marker of chondrocytes in the resting zone of the growth plate (23, 32). These findings show that FB2 is highly responsive during adaptive remodeling. Additionally, results from immunofluorescence staining were consistent with our bioinformatic analyses. APOE-positive cells significantly increased and presented higher proliferative properties within the RT from ADD and CADD groups compared with the sham group (Figure 2, J and K, and Supplemental Figure 5).

Additionally, we analyzed gene expression changes between the experimental (ADD+CADD) RT group and sham RT group. Compared with the sham group, FBs from experimental group showed alteration in genes related to ECM organization and turnover (*COL3A1*, *WISP2*, *COMP*, *FMOD,* and *CST3*) (33, 34). Concurrently, genes associated with FB proliferation and apoptosis (*BTG2* and *IFI6*) were also differentially expressed (Supplemental Figure 6A) (35, 36).

Specifically, FBs from the CADD RT group were characterized by ECM remodeling–related genes (*COL3A1*, *COL11A1,* and *FMOD*), indicating enhanced ECM synthesis and reorganization. Fibroblasts from the ADD RT group exhibited a stress-activated profile (*CCL2*, *CCL19*, and *SOD2*) (37), suggesting injury-induced inflammation and compensatory repair processes. In contrast, FBs from the sham RT group predominantly expressed genes involved in maintaining ECM homeostasis and progenitor cell recruitment (*CXCL12*, *CST3,* and *COMP*) (Supplemental Figure 6B) (34, 38). Collectively, these findings support a transcriptional shift toward an activated ECM remodeling–related FB phenotype following ADD.

### A specific ECM-related MC subcluster emerges in RT after ADD induction.

**Figure 3:**
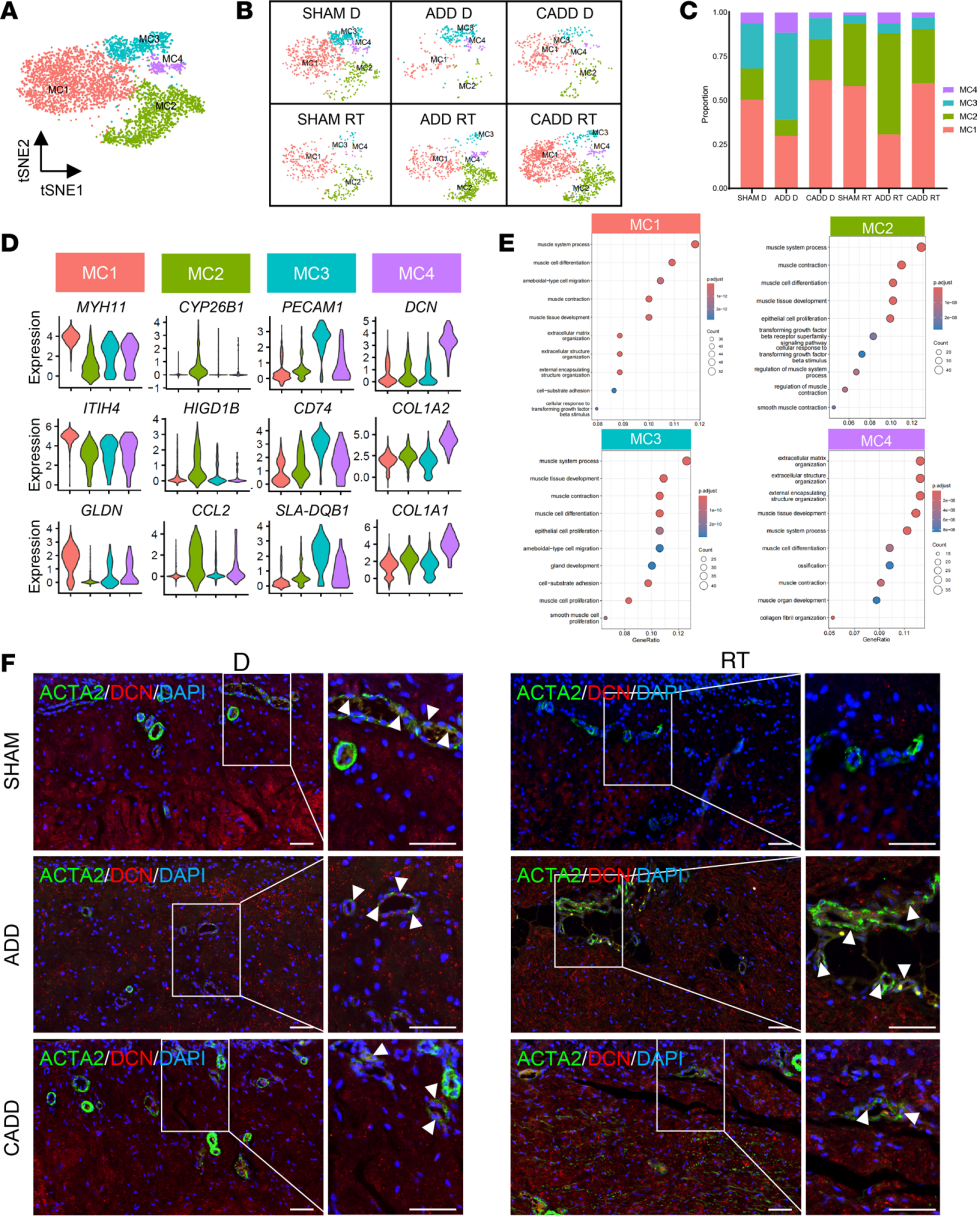
Identification of a specific ECM-related MC subcluster in the RT after ADD induction. (**A**) Dimension reduction presentation of combined single-cell transcriptome data of mural cell (MC) cluster from TMJ disc and RTs of all groups. Four subclusters of MCs were identified and visualized by a t-distributed stochastic neighbor embedding (t-SNE) plot. (**B**) Dimension reduction presentation of single-cell transcriptome data of MC cluster in sham, ADD, and CADD groups from TMJ disc and RTs via t-SNE, displayed separately by tissue origins and groups. (**C**) Bar graph shows the fraction of MC subtypes by tissue origins and groups. (**D**) Violin plots show the expression of differentially expressed genes (DEGs) in different MC subclusters. Expression values are normalized. (**E**) Dot plot of Gene Ontology (GO) Biological Process enrichment analysis of DEGs in different MC subclusters. (**F**) Representative images of TMJ discs and RTs subjected to immunofluorescence staining for ACTA2 and DCN in different groups; 3 random fields were selected from each of the 2 pig samples. Green, ACTA2; red, DCN; blue, DAPI; white arrowhead, cells coexpressing ACTA2 and DCN. Scale bars: 50 μm.

MCs are essential components of blood vessels, which attach to the surface of vascular ECs and maintain vasculature stability (39). Some studies have also suggested their pivotal roles in fibrosis (40). Among four MC subclusters, MC1, MC3, and MC4 displayed high expression of *ACTA2*, *CNN1,* and *MYH11*, indicating a smooth muscle cell (SMC) identity; MC2 was identified as a pericyte by its high expression of *HIGD1B*, *RGS5,* and *NUDUFA4L2* (Figure 3, A and B and Supplemental Figure 7, A and B). We further defined MC1 as a typical SMC due to its high expression of *MYH11* and *ACTA2*. MC3 was named as an immune-related SMC with its expression of *CD74*, and MC4 was considered to be an ECM-related SMC owing to its specific expression of *DCN*, *COL1A1,* and *COL1A2* and its biological association with ECM organization (Figure 3, D and E). Interestingly, MC4 was nearly absent in the RT from the sham group but increased in the ADD and CADD groups (Figure 3C). Pseudo-time trajectory analysis revealed that MC4 originated from the pericyte (MC2), and subsequently differentiated into a typical SMC (MC1) and immune-related SMC (MC3) (Supplemental Figure 7, C and D). The absence of MC4 in RT from the sham group and presence of MC4 in RT from ADD and CADD groups were further confirmed using immunofluorescence staining (Figure 3F).

We further evaluated subtle transcriptional changes in other cell clusters after the onset of ADD. Four EC subclusters were identified as vein ECs, developmental ECs, artery ECs, and lymphatic ECs (Supplemental Figure 8A). The developmental ECs with high angiogenesis potential decreased in RT of the ADD and CADD groups, suggesting inhibited angiogenesis during tissue remodeling (Supplemental Figure 8B) (41). With regard to immune cells, they were more abundant and diverse in the RT than the disc, indicating that the RT may be more immunologically active. The percentages of macrophages and neutrophils increased in RT after ADD (Supplemental Figure 8, C and D).

### Putative signaling network for the intercellular crosstalk regulating adaptive remodeling of the RT.

To probe differences in cell-cell communication between the sham RT and ADD RT groups, as well as between the sham RT and CADD RT groups, we employed comparative CellChat. A total of 13 cell clusters, including ECs, immune cells, cells in the cell cycle, and subclusters of FBs and MCs were combined for analysis. Network centrality analysis was performed by computing the outgoing and incoming interaction strengths of each cell subpopulation to assess their likelihood as signaling sources and targets, respectively. In the sham RT group, interactions between FB subclusters (mainly FB1 and FB3) and other cells were most intense. In contrast, the newly emerged MC4 subcluster showed drastically increased interactions with other cells after ADD (Supplemental Figure 9B). We further compared the information flow, defined by the sum of communication probability among all pairs of cell groups in the inferred network (Supplemental Figure 9C). Pathways including MK, PERIOSTIN, FGF, BMP, TWEAK, SPP1, IL6, and MIF were exclusively active in both the ADD RT and CADD RT groups, indicating enhanced cell proliferation, migration, and differentiation (42–47). Other pathways such as GAS, PDGF, and VISFATIN revealed decreased information flow after ADD induction, which are known to be associated with FB inhibition, vascular development, and inflammatory responses (48–50).

**Figure 4:**
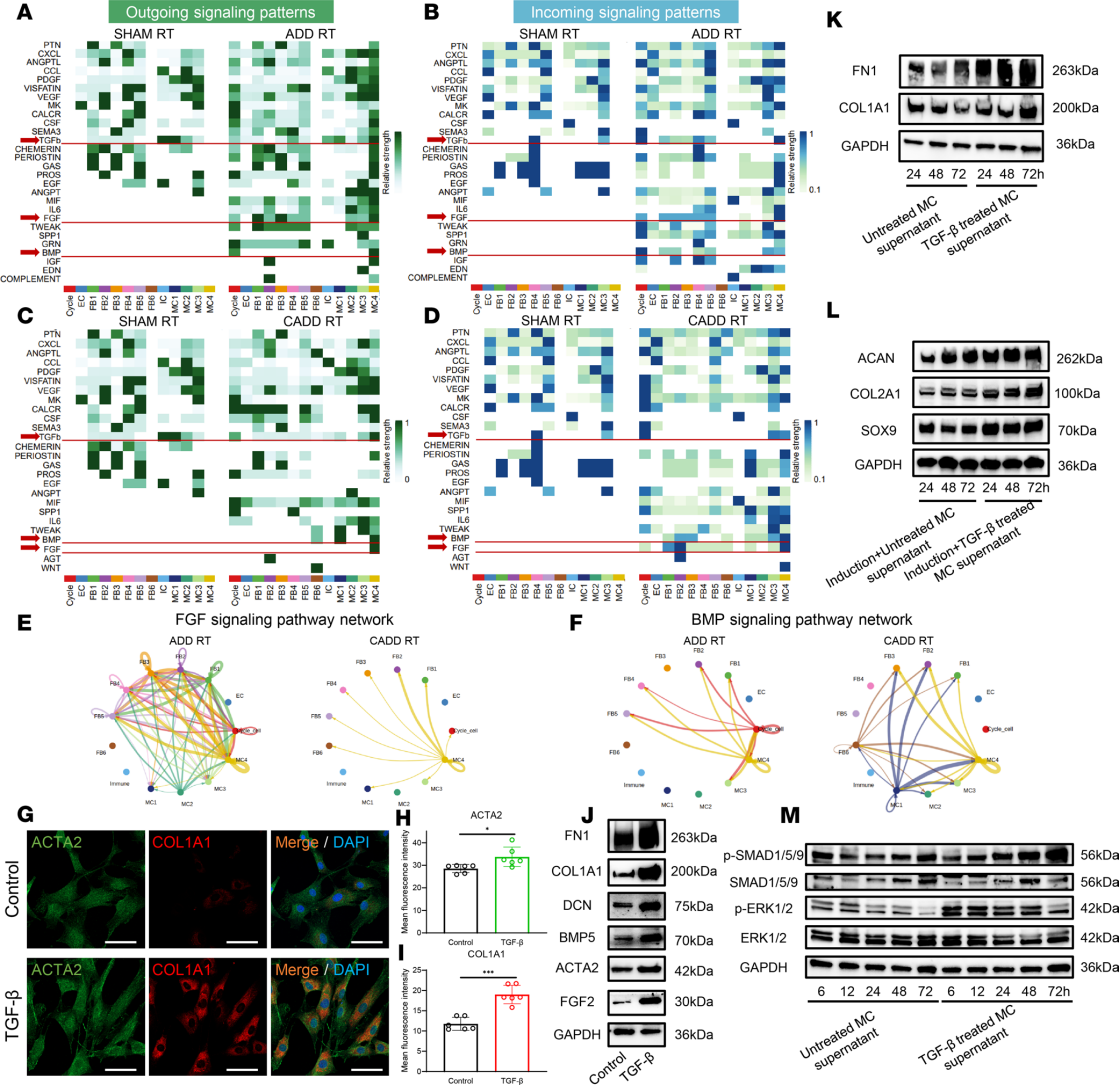
FGF and BMP signaling pathways are involved in adaptive remodeling of the RT. Heatmap of outgoing signaling pathways in different cell clusters in sham and ADD RT groups (**A**) and sham and CADD RT groups (**C**). Heatmap of incoming signaling pathways in different cell clusters in sham and ADD RT groups (**B**) and sham and CADD RT groups (**D**). The inferred FGF signaling pathway (**E**) and BMP5 signaling pathway (**F**) networks in ADD and CADD RT groups. Edge width represents the communication probability between cell types. (**G**) Immunofluorescence staining for ACTA2 and COL1A1 in mural cells (MCs) and quantification (**H** and **I**) (*n* = 6). Statistical significance was determined by Student’s *t* test. Data represent mean ± SD. *P < 0.05, ***P < 0.001. Green, ACTA2; red, COL1A1; blue, DAPI. Scale bars: 50 μm. (**J**) Evaluation of FB and SMC marker levels in MCs. (**K**) Evaluation of fibrogenic protein levels in FBs treated with different MC supernatants. (**L**) Evaluation of chondrogenic protein levels in FBs treated with different MC supernatants under chondrogenic induction. (**M**) Evaluation of SMAD1/5/9 and ERK1/2 cascade activation in FBs treated with different MC supernatants. For representative Western blot, 3 replicates were conducted.

Next, we investigated the candidate pathways underlying the changes in cellular compositions and tissue features after ADD induction. The newly emerging MC4 cluster exhibited extensive potential for both self- and reciprocal interactions in the ADD RT and CADD RT groups, such as TGF-β, FGF, and BMP pathways (Figure 4, A–D). We speculated that MC4 may be an important regulator of tissue remodeling. Active FB-MC4 interactions were also found, particularly enriched in FGF and BMP signaling (Figure 4, E and F). In the ADD RT group, FGF signaling exhibited intensive exchanges between FBs and MCs, whereas BMP signaling was mainly sent by MC4 and received by FB subclusters. In the CADD RT group, MC4 presented as the only sender of FGF signaling, and all FB subclusters were considered as receivers. Notably, interaction between FB2 and MC4 via FGF signaling was particularly intense. BMP signaling was sent by MC1 and MC4, while received mainly by MC4, FB2, and FB3 (Figure 4, E and F). Specifically, the receptor-ligand signaling communication probabilities from MC4 to FBs showed that FGF2-FGFR1 and BMP5-BMPRA1+BMPR2 are the major contributors in FGF signaling and BMP signaling, respectively (Supplemental Figure 10). Immunofluorescence staining demonstrated markedly increased expression of FGF2 and BMP5 in MCs within the RT of the ADD and CADD groups (Supplemental Figures 11 and 12), supporting the predicted signaling activity in vivo.

In vitro functional experiments showed that TGF-β treatment markedly induced transformation of MCs into MC4, as indicated by enhanced expression of FB (FN1, COL1A1, and DCN) and SMC (ACTA2) markers (Figure 4, G–J). Consistently, TGF-β–induced transition of MCs toward the MC4 phenotype resulted in elevated FGF2 and BMP5 levels (Figure 4J). In addition, conditioned medium from MC4 enhanced ECM deposition and chondrogenic marker expression in FBs (Figure 4, K and L). Consistent with our in vitro findings, FGF2 and BMP5 treatment exhibited synergistic effects on enhancing ECM synthesis and chondrogenic differentiation of FBs (Supplemental Figure 13).

To reveal the underlying mechanism, we evaluated changes of classical pathways activated by FGF2 and BMP5, including the ERK1/2 and SMAD1/5/9 cascades. We observed the activation of the ERK1/2 pathway as early as 6 hours after stimulation, followed by the activation of the SMAD1/5/9 cascade about 24 hours after stimulation (Figure 4M). These results indicate that MC4 induces sequential activation of distinct pathways to orchestrate the phenotypic transition of FBs. The early activation of ERK1/2 signaling likely drives FB proliferation and ECM deposition, initiating RT remodeling. As remodeling progresses, the SMAD1/5/9 signaling become progressively enhanced, promoting ECM reorganization and inducing fibrocartilage formation.

### Selection of candidate compounds enhancing remodeling capability of FBs.

To identify drugs that enhance FB remodeling, we focused on predicted drug responses associated with feature genes from FB subsets. We combined top-ranked genes from a pathway-aware multilayered hierarchical network (P-NET) and Seurat with the medicine database (ConnectivityMap and DGIdb) to look for candidate compounds. The screening workflow is shown in the schematic diagram (Supplemental Figure 14).

Molecular profiles of FBs by comparing the sham RT and ADD RT and the sham RT and CADD RT groups were fed into P-NET, which maps genes onto a hierarchical network of weighted nodes (51). We visualized the top 10 most important nodes using Sanky diagrams to reveal the interaction between different features, genes, pathways, and biological processes and to study the paths of impact from the input to the outcome (Supplemental Figure 15, A and B). Highly ranked genes in both ADD and CADD groups included *CST3*, *FGL2,* and *B2M*. *CST3* encodes cystatin C, a cysteine protease inhibitor that promotes matrix deposition and is overexpressed in cardiac, lung, and liver fibrosis (34, 52). Fibrinogen-like protein 2 (FGL2) regulates the polarization of macrophages (53) and is enriched in patients with ADD without osteoarthritis compared with patients with osteoarthritis (54). *B2M* encodes β2-microglobulin, a serum protein with antibacterial activity (55). After ADD induction, processes relating to metabolism of proteins, gene expression, metabolism of RNA, and the cell cycle supported adaptive remodeling as a condition with large systemic effects on metabolic, immune, and cellular homeostasis. In FBs from the CADD RT group, *COL1A1*, *COL1A2,* and *COL3A1* ranked in the top 10 and were associated with collagen formation and ECM organization, consistent with histological observations that CADD RT exhibited superior tissue remodeling compared with ADD RT.

Five candidate compounds were identified after scoring with the gene expression feature data from the medicine database (Supplemental Table 2). According to their mechanism of action, potential targets, and score ranking, 3 molecules (batimastat, sunitinib, and zaprinast) were selected for in vitro testing. Batimastat is a broad-spectrum peptide inhibitor of MMPs, which exerts therapeutic effects in heart failure and pulmonary fibrosis by MMP inhibition (56, 57). Sunitinib is a tyrosine kinase inhibitor targeting VEGF and PDGFR receptors. It shows notable efficiency in advanced renal cell carcinoma and intolerant gastrointestinal stromal tumor treatment (58) but may induce side effects such as cardiac fibrosis (59). Zaprinast is a phosphodiesterase 5 (PDE5) inhibitor and G protein–coupled receptor 35 (GPR35) agonist, targeting inflammation and pain reduction (60, 61). However, reports on its activity in the regulation of ECM remodeling are currently lacking.

Cytotoxicity of the selected compounds was assessed using CCK-8 assays. Batimastat (1–100 nM) and zaprinast (0.1–100 μM) possessed good biocompatibility to porcine FBs, whereas sunitinib exhibited significant cytotoxicity even at 1 nM (Supplemental Figure 15, C–E). We further determined the optimal drug concentration by assessing the expression of target genes (Supplemental Figure 15, F–H). In the following experiments, 10 nM batimastat, 1 nM sunitinib, and 1 μM zaprinast were used.

**Figure 5:**
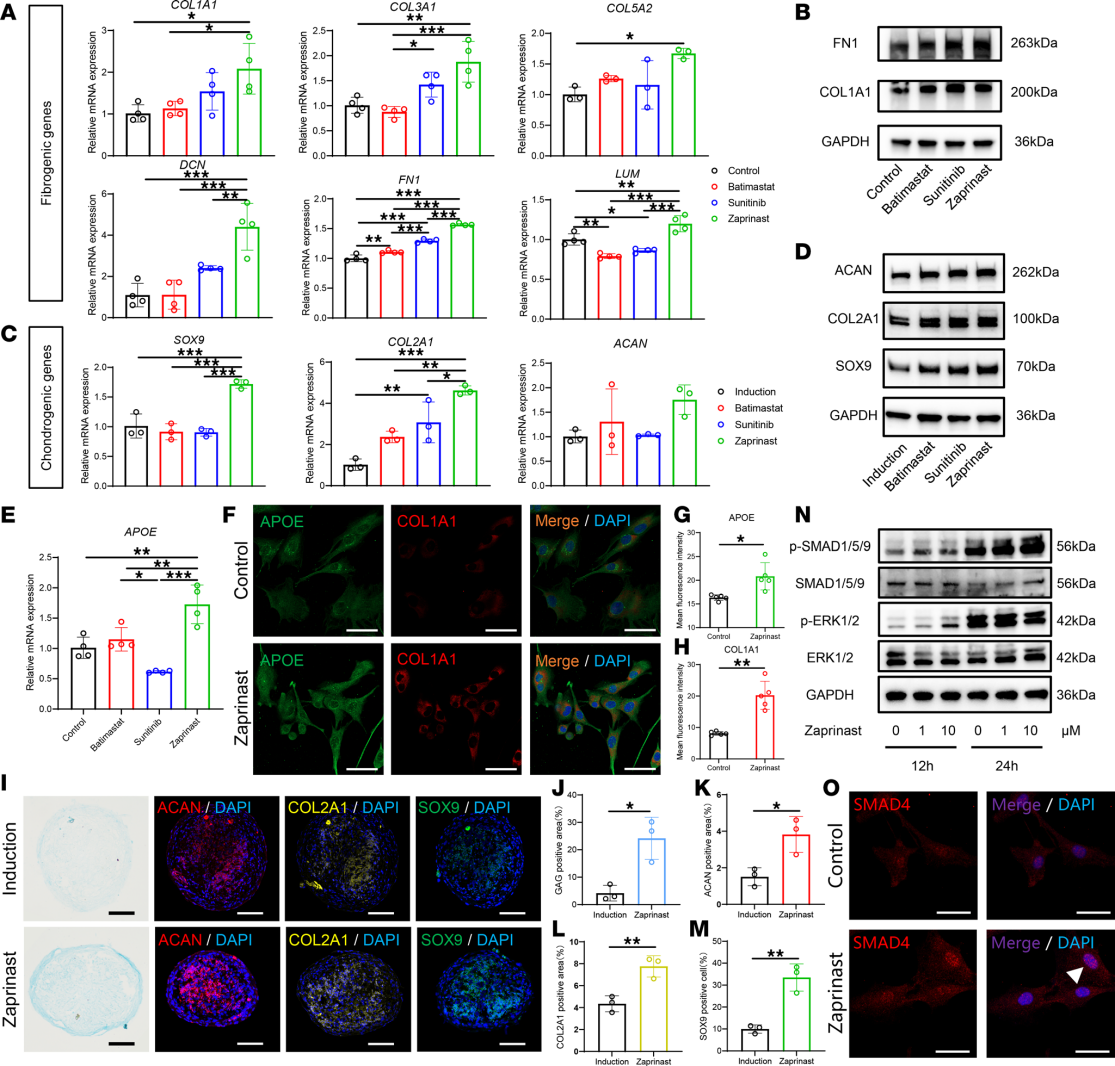
Zaprinast exhibits most prominent profibrotic and chondrogenic effects on FBs by ERK1/2 and SMAD1/5/9 cascade activation. Evaluation of fibrogenic gene expression (**A**) and protein levels (**B**) treated with batimastat, sunitinib, and zaprinast. Evaluation of chondrogenic gene expression (**C**) and protein levels (**D**) treated with batimastat, sunitinib, and zaprinast under chondrogenic induction. (**E**) Gene expression of *APOE* treated with batimastat, sunitinib, and zaprinast. Fold-changes were normalized with *GAPDH* (*n* = 3–4). Statistical significance was determined by 1-way ANOVA. Data represent mean ± SD. *P < 0.05, **P < 0.01, ***P < 0.001. For representative Western blot, 3 replicates were conducted. (**F**) Immunofluorescence staining for APOE and COL1A1 in FBs and quantification (**G** and **H**) (*n* = 5). Statistical significance was determined by Student’s *t* test. Data represent mean ± SD. *P < 0.05, **P < 0.01. Green, APOE; red, COL1A1; blue, DAPI. Scale bars: 50 μm. (**I**) Alcian blue staining and immunofluorescence staining (ACAN, COL2A1, and SOX9) of pellet culture and quantification (**J**–**M**) (*n* = 3). Statistical significance was determined by Student’s *t* test. Data represent mean ± SD. *P < 0.05, **P < 0.01. Red, ACAN; yellow, COL2A1; green, SOX9; blue, DAPI. Scale bars: 100 μm. (**N**) Evaluation of ERK1/2 and SMAD1/5/9 cascade activation in FBs treated with zaprinast. For representative Western blot, 3 replicates were conducted. (**O**) Immunofluorescence staining displays nuclear localization of SMAD4 in FBs treated with zaprinast (white arrowhead). Red, SMAD4; blue, DAPI. For representative immunofluorescence staining of SMAD4, 3 replicates were conducted. Scale bars: 50 μm.

We investigated the effect of these compounds on ECM synthesis and chondrogenesis in FBs. Western blot and qRT-PCR analyses revealed that sunitinib and zaprinast markedly increased ECM gene expression, whereas batimastat had minimal effect (Figure 5, A and B). Under chondrogenic induction, FBs treated with zaprinast showed the highest expression of chondrogenic differentiation markers compared with batimastat and sunitinib (Figure 5, C and D). As shown by immunofluorescence staining, zaprinast significantly increased the expression of COL1A1 and APOE in FBs (Figure 5, E–H). Notably, elevated APOE expression indicates enhanced remodeling capacity in FBs, a characteristic feature of the FB2 phenotype. Alcian blue staining and immunofluorescence staining (SOX9, ACAN, COL2A1) of pellet culture under chondrogenic differentiation further confirmed its effects on chondrogenesis (Figure 5, I–M). Mechanistically, zaprinast promoted fibrocartilage differentiation of FBs through activating the ERK1/2 and SMAD1/5/9 pathways, the canonical downstream pathways of FGF2 and BMP5 (Figure 5, N and O). In summary, zaprinast appeared to have optimal effects in enhancing ECM synthesis and chondrogenic differentiation of FBs.

### Zaprinast alleviates disc and RT deformation and mitigates condyle damage in vivo.

**Figure 6:**
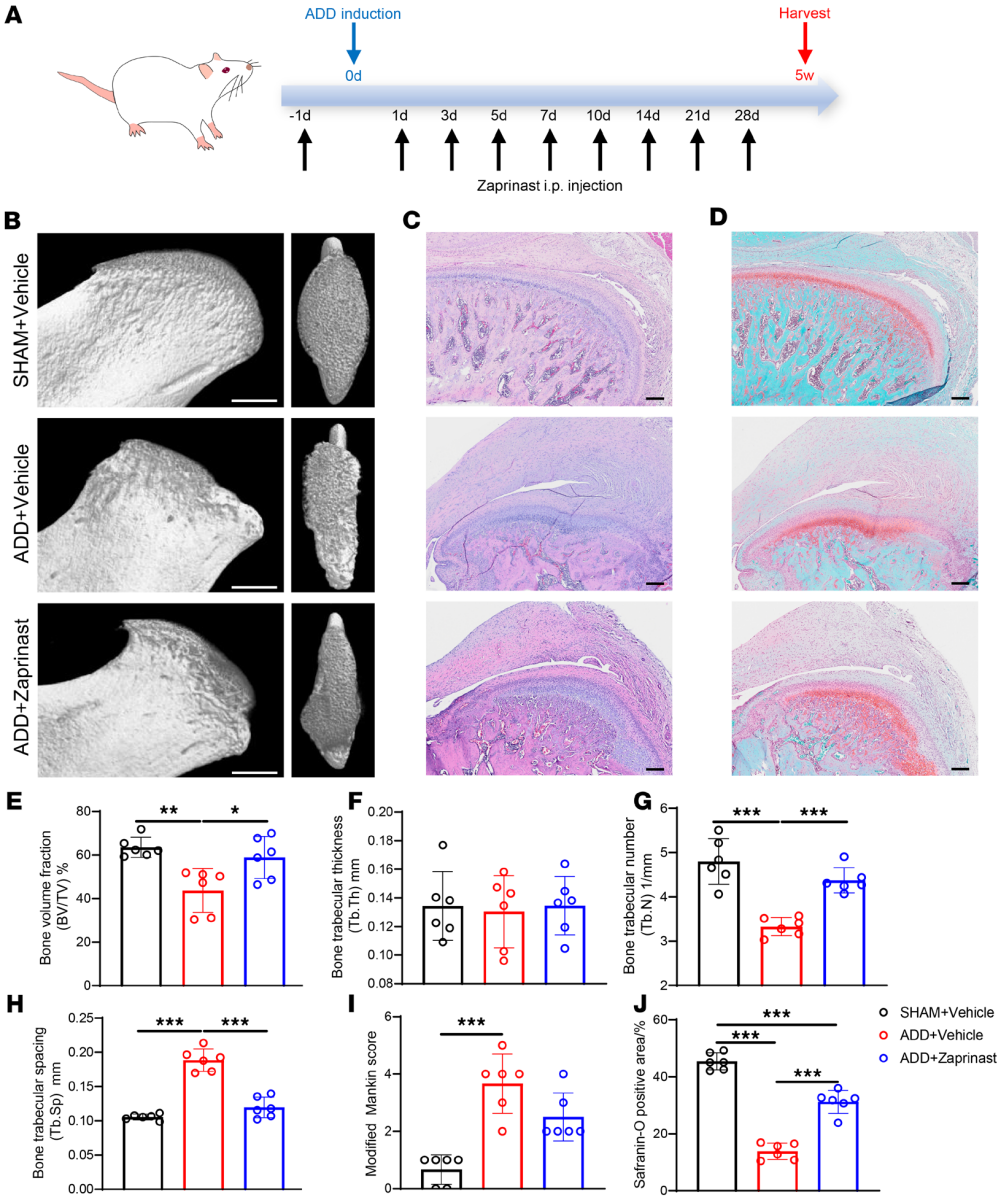
Zaprinast alleviates deformation of the disc and RT and damage of the condyle in vivo. (**A**) Timeline of ADD induction and zaprinast administration in a rat model. (**B**) Representative 3D reconstruction images of condyle from Sham+Vehicle, ADD+Vehicle, and ADD+zaprinast groups. Scale bars: 1 mm. (**C**) H&E staining showing alteration in configuration of disc and RT. Scale bars: 200 μm. (**D**) Safranin O and fast green staining showing changes in proteoglycan content. Scale bars: 200 μm. (**E**–**H**) Quantitative analysis of condyle profiles (*n* = 6). (**I**) Mankin score evaluation (*n* = 6). (**J**) Quantitative analysis of the proportion of safranin O–positive areas (*n* = 6). Statistical significance was determined by 1-way ANOVA or Kruskal-Wallis test. Data represent mean ± SD. *P < 0.05, **P < 0.01, ***P < 0.001.

We investigated whether zaprinast could promote adaptive remodeling of the RT and maintain condyle integrity after ADD in vivo. Zaprinast was i.p. administered before and after ADD induction (Figure 6A). In the Sham+Vehicle group, the disc remained thin and well-oriented. Five weeks after ADD induction, the ADD+Vehicle group exhibited alterations including thickening and anterior displacement of the disc, disorganized collagen architecture in the posterior band and RT, and inflammatory cell infiltration. These pathological changes were partially alleviated by zaprinast treatment, which exhibited improved orientation and organization of collagen fibers (Figure 6, C and D). During the observation period, no perforations or tears were observed in the disc or RT in any group. We did not observe fibrocartilage formation in the rat model, likely reflecting species-specific differences in masticatory patterns and TMJ anatomy.

We further assessed changes in condylar cartilage and subchondral bone. H&E staining revealed hyperplasia of the superficial fibrous layer and irregularities of the cartilage in the ADD+Vehicle group compared with the Sham+Vehicle group. Consistently, safranin O and fast green staining demonstrated substantial loss of cartilage matrix in the ADD+Vehicle group. Micro-CT analysis revealed condylar flattening (Figure 6B), reduced bone volume fraction, decreased trabecular number, and increased trabecular spacing (Figure 6, E–H). Zaprinast treatment mitigated degenerative changes in cartilage morphology (Figure 6I), increased cartilage matrix content (Figure 6J), and partially restored subchondral bone morphology (Figure 6, E–H).

In summary, these findings indicate that zaprinast partially restores the ordered structure of the disc and RT and concurrently attenuates cartilage matrix loss and subchondral bone resorption after ADD induction.

## Discussion

Clinical observations indicate that most temporomandibular disorder cases follow a mild and fluctuating course. In patients with ADD without reduction, the natural course is that their clinical symptoms tend to alleviate, with improvements in mandibular movement and masticatory efficiency (62), suggesting that TMJ has a remarkable adaptive capacity. After ADD, mechanical load shifts from the articular disc to the mechanically inferior RT. Finite element analysis has confirmed that perforations in the retrodiscal region show higher maximum equivalent stress than perforations in other areas, explaining why perforation predominantly occurs at the RT (63). Moreover, retrodiscal attachment dissection hastens ADD progression (64), whereas adaptive remodeling correlates with condylar cartilage recovery (9). These findings underscore the importance of RT integrity and remodeling for TMJ stability.

Our UADD porcine model revealed bilateral RT adaptation, highlighting the need to monitor both affected and contralateral joints in the clinic. ScRNA-Seq analysis revealed cellular heterogeneity between the TMJ disc and RT, identifying key cell subpopulations driving remodeling. Cell-cell communication analysis implicated FGF and BMP signaling as critical regulators. Using biologically informed deep learning, we identified zaprinast as a potential therapeutic compound that promotes adaptive remodeling of RT and prevented TMJ damage at the early stage of ADD.

Although remodeling of RT after ADD has been recognized for decades, detailed cellular mechanisms have remained unclear due to technical limitations. ScRNA-Seq analysis provided a systematic view of cell diversity between the TMJ disc and RT, revealing dynamic changes after ADD. In pigs that underwent sham operation, the RT exhibited a cell composition broadly similar to that of the disc but with distinct relative proportions, supporting its inherent potential for disc-like transformation. Notably, a developmentally potent FB2 subpopulation increased after ADD. We identified several features of FB2 that may drive remodeling. FB2s predominantly express resident progenitor cell marker genes *APOE* and *EFEMP1* (23, 32). Pseudo-time trajectory analysis revealed that FB2 could differentiate into ECM-producing FBs (FB1, FB3) and modulatory FBs (FB4–6). FB3, in particular, displayed fibrocartilaginous characteristics by predominant expression of *COL1A1*, *COL1A2*, *ACAN,* and *COMP*. Histology evaluation confirmed GAG accumulation in RT after ADD, supporting mechanical-induced adaptation (6). These findings suggest that FB2 is important for FB expansion and ECM deposition during adaptive remodeling.

Apart from the reparative role of FBs, the supporting role of minor cell populations is also noteworthy. ScRNA-Seq analysis and lineage tracing studies verified that pericytes transiently express FB markers and contribute to vascular maturation after myocardial infarction (40). Similarly, a new mural cell subtype (MC4) emerged after ADD, displaying ECM-related signatures and broad interactions with other cells. TGF-β predominantly participated in MC4 self-interaction, aligning with its profibrotic role in promoting FB proliferation, ECM synthesis, and myofibroblast differentiation (65). In vitro, TGF-β treatment induced the transition of MCs toward an MC4-like phenotype. Conditioned medium derived from MC4-like cells promoted ECM synthesis and chondrogenic differentiation of FBs, further supporting a paracrine role of MC4 in FB activation.

Abnormal loading can induce fibrocartilaginous metaplasia and ligament ossification via Hedgehog and BMP signaling (66, 67). We identified increased pathways in the ADD RT and CADD RT groups including PERIOSTIN, FGF, BMP, TWEAK, SPP1, and IL6, which are closely related to fibrosis (43–46). Notably, FGF and BMP signaling were engaged in MC4 to FB interactions, promoting FB expansion and chondrogenic differentiation. These findings are consistent with observations in fibrotic osteoarthritis synovial tissues, where FGF and BMP drive ECM remodeling, contrasting with inflammatory phenotypes dominated by predominant inflammatory cytokine expression and pain (68). MC4-derived signals modulate FB behavior through coordinated activation of ERK1/2 and SMAD1/5/9 pathways. FGF2 is a well-established activator of ERK1/2, and ERK activation can indirectly potentiate BMP/SMAD1/5/9 signaling (69) and directly phosphorylate SMAD4 (70). BMP5 activates both canonical SMAD and noncanonical MEK/ERK pathways in developmental and neural crest contexts (71). These observations suggest that MC4-derived FGF2 and BMP5 may act synergistically to amplify ERK1/2/SMAD1/5/9 signaling, thereby promoting FB remodeling.

By combining a causal-inspired (P-NET) approach with a correlation-based (Seurat) approach, we identified high-confidence candidate targets that support tissue adaptation during ADD, which are both statistically correlated and biologically interpretable. Five potential compounds were identified, with zaprinast showing superior efficacy in enhancing FB remodeling. Furthermore, zaprinast partly restored TMJ deformation in a rat ADD model, providing in vivo support for the therapeutic potential of zaprinast. Previous studies have reported the osteogenic potential of zaprinast by promoting angiogenesis and BMP synthesis (72). Similarly, another PDE inhibitor, sildenafil, potentiates BMP signaling in pulmonary arterial SMCs by enhancing BMP4-induced phosphorylation of SMAD1/5 (73). Mechanistically, our study revealed that zaprinast promoted fibrocartilage differentiation of FBs via the ERK1/2 and SMAD1/5/9 pathways, which are canonical downstream of FGF2 and BMP5.

Given the limited availability of early-stage human ADD tissues, we employed a porcine model for its anatomical and physiological similarity to humans. Nevertheless, cross-species validation is essential, including validation of key cell subsets and pathways in human TMJ tissues and functional assessment in human-derived models. Pilot observational studies in humans could further determine whether the identified molecular signatures correlate with better clinical parameters. Long-term follow-up in patients with non-reducing ADD and studies in animal ADD models indicate that RT remodeling can serve as a beneficial process, leading to improved joint function (9–11). Nevertheless, dysregulated fibrosis may become maladaptive, contributing to joint stiffness and pain. Our findings suggest that MC4-mediated remodeling may initially support mechanical compensation, but future longitudinal studies are needed to determine whether it remains beneficial or transitions to pathological fibrosis.

Additionally, technical limitations include potential cell-type bias during tissue dissociation, which may underrepresent vulnerable populations such as adipocytes. Reduced adipose content in RT may result from inhibited adipogenesis (74), lineage reprogramming of adipocytes into myofibroblasts (75), or stress-driven adipocyte apoptosis (76). Future studies using single-nucleus RNA-Seq combined with functional validation are needed to clarify the contributions of adipocytes. The potential role of neuronal paracrine signaling in RT remodeling also warrants future investigation.

In conclusion, our findings demonstrated that FB activation is pivotal for early RT adaptation after ADD and is driven by the emergence of an ECM-remodeling MC subcluster. We implicate FGF and BMP signaling as key regulators of enhanced fibrosis and chondrogenic differentiation of FBs by sequential activation of ERK1/2 and SMAD1/5/9 cascades. Although current treatments for ADD lack targeted approaches for TMJ adaptation, our data suggest zaprinast may be used as a promising drug for conservative treatment of ADD.

## Methods

### Sex as a biological variable.

Our study examined female animals considering that temporomandibular disorder onset is more common in young women, potentially due to differences in hormonal factors, pain sensitivity, and health-seeking behavior (77). Therefore, we utilized 5-month-old female Bama miniature pigs and 10-week-old female Sprague-Dawley rats that were considered sexually mature. Although we selected female pigs and rats in this study, we anticipate the fundamental mechanisms are applicable to both sexes.

### Animals and groups.

Four female Bama miniature pigs aged 5 months old and 18 female Sprague-Dawley rats aged 10 weeks old were purchased from Zhejiang Chinese Medical University Laboratory Animal Research Center. Two pigs were randomly assigned to undergo sham operation on the left TMJ (sham group), and the right TMJ remained untreated. The other 2 pigs underwent ADD surgery on the left TMJ (ADD group), and the contralateral right TMJ was left untreated (CADD group). Rats were randomly divided into 3 groups: Sham+Vehicle group (*n* = 6), ADD+Vehicle group (*n* = 6), and ADD+zaprinast group (*n* = 6). Zaprinast (MCE) was dissolved in solvent (10% DMSO + 40% PEG 300 + 5% Tween-80 + 45% saline) and i.p. injected 1 day before surgery and 1, 3, 5, 7, 10, 14, 21, and 28 days after surgery (dosage 10 mg/kg; ref. 78). An equivalent volume of solvent was administrated in vehicle groups.

### Generation of UADD model.

The surgical methods to generate the UADD porcine model were modified according to a previous study of rabbit models (16). After i.v. anesthesia, the left preauricular region was shaved and disinfected. A 4 cm curved incision was made in line with the lateral canthus of the eye. After incising through subcutaneous tissue and periosteum, we exposed the zygomatic arch and TMJ capsule. The inferior joint space was carefully opened by dissecting the lateral capsule without damaging the TMJ disc or articular cartilage surface. The anterior and lateral attachments were partially released while preserving the posterior attachment.

Subsequently, an orthodontic mini-implant was screwed into the zygomatic arch approximately 2 cm from its posterior margin. Stainless steel was used to connect the mini-implant and a nickel-titanium spring. To avoid osseointegration, a titanium-reinforced e-PTFE nonabsorbable membrane was placed between the zygomatic arch and nickel-titanium spring. After that, we penetrated the anterior attachment of the articular disc with a 4-0 nylon suture and knotted it to the posterior end of the nickel-titanium spring. By extending the spring from 4 mm to 14 mm, we applied 1 N of tension to displace the disc anteriorly, although immediate displacement was not observed. Finally, the wound was thoroughly irrigated with saline and closed in layers with 3-0 Monocryl sutures.

Similarly, rats were anesthetized with pentobarbital sodium (50 mg/kg i.p.), and the left preauricular region was shaved and sterilized. After local anesthesia with lidocaine hydrochloride (2%), the zygomatic arch and lateral TMJ capsule was carefully opened to expose the inferior joint space. A hole was carefully drilled at the front junction of the zygomatic arch with a 0.8 mm round bur. Orthodontic elastic bands (1/8, 3.5 oz, 3M Unitek) were used to draw the disc forward. One end of the elastic band was attached to a 5-0 nylon suture, which penetrated through the anterior band of the disc. Then, the other end was attached to the hole. By elongating the elastic band from 3 mm to 10 mm, a tension force of 1 N was generated to stretch the disc anteriorly. The wound was thoroughly irrigated with saline and closed in layers with a 5-0 nylon sutures.

Postoperatively, animals received i.m. antibiotics for 3 days. No notable differences in diet or weight were observed between the groups, and all animals survived the study period. Five weeks after surgery, pigs were euthanized via bloodletting under deep anesthesia. Rats were also euthanized 5 weeks after surgery. Subsequent gross examination confirmed the ADD of the disc in the ADD group specimens.

### Single-cell digestion and sequencing.

First, we dissected the disc and RT from the condyle. Next, tissues (5 × 5 × 5 mm^3^) were cut from the intermediate region of the disc (disc sample) and RT near the posterior band (RT sample). Specimens were cut into small pieces (2–3 mm), and then digested with 4 mg/mL Pronase (Roche) for 1 hour, followed by digesting with 2 mg/mL collagenase P (Roche) in FBS-free high-glucose DMEM (Gibco) for 2 hours. After filtering with 70 μm and 40 μm filters, the cell suspension was loaded into the 10x Chromium Single Cell Platform (10x Genomics). Generation of gel beads in emulsion (GEMs), barcoding, GEM-RT clean-up, cDNA amplification, and library preparation and quantification were all performed according to the manufacturer’s protocol (Single Cell 3′ library and Gel Bead kit v3). The final library pool was sequenced on the Illumina HiSeq sequencing systems.

### Quality control of scRNA-Seq data.

This study included scRNA-Seq data from porcine-derived samples (Sham D, ADD D, CADD D, Sham RT, ADD RT, and CADD RT), which were processed using the Seurat R package (v4.0.5). The raw gene expression matrices were obtained from Cell Ranger output directories and imported using the Read10X function. For each sample, a Seurat object was created with the CreateSeuratObject function, and basic quality control metrics such as gene count distributions were initially assessed using basic R functions (e.g., summary, dim).

To further eliminate low-quality cells, we calculated the proportion of mitochondrial gene expression (e.g., *ND1*, *ND2*, *COX1*, *CYTB*) for each cell based on a curated mitochondrial gene list and retained only cells with less than 10% mitochondrial gene content (percent.mt). Furthermore, outlier cells were identified and removed using the median absolute deviation method applied to the log_10_-transformed total UMI counts (nCount_RNA) and the number of detected genes (nFeature_RNA). Cells outside the median ± 3 × median absolute deviation range were excluded. At the gene level, after cell-level quality control, we reconstructed the expression matrix by retaining only genes expressed in at least 2 cells (min.cells = 2) to filter out low-abundance or biologically irrelevant noise genes.

### Preprocessing and integration of scRNA-Seq data.

Each Seurat object was independently normalized using the NormalizeData function. Highly variable genes were identified using the variance-stabilizing transformation method via FindVariableFeatures, with the top 2,000 most variable genes selected for downstream integration. To correct for batch effects and merge the datasets, we employed the canonical correlation analysis–based integration pipeline in Seurat. Specifically, the FindIntegrationAnchors function was used to identify anchor pairs across samples using the top 15 principal components, and the IntegrateData function was then applied to generate a batch-corrected, integrated Seurat object.

### Clustering, annotation, and trajectory analysis of scRNA-Seq data.

The integrated dataset was subjected to dimensionality reduction, clustering, and differential gene expression analysis using Seurat (v4.0.3.1). Clusters were manually annotated based on known marker genes of cell types (Supplemental Figure 1D). Specific cell populations such as FBs and MCs were extracted for refined subclustering and re-annotation based on established literature. Functional enrichment analysis of marker gene lists was conducted using Metascape for GO term interpretation. Cell-cell communication analysis was performed using CellChat (v1.5.0), and trajectory inference was conducted using Monocle (v2.30.1) and CytoTRACE (v0.3.3).

### Histological evaluation.

Dissected specimens were fixed in 4% paraformaldehyde (PFA) (pH 7.4, Servicebio) at room temperature overnight, embedded in paraffin after dehydration, and sectioned into 5 μm thick slices. Condyle samples were decalcified with 0.5 M EDTA (pH 7.2, Servicebio) for 3 months before embedding. Representative sections were stained with H&E (Fdbio Science), Alcian blue staining (Beyotime), and safranin O and fast green staining (Solarbio) according to the manufacturer’s instructions. The safranin O–positive area was calculated with ImageJ (NIH) software (v1.54). A modified Mankin score was used for evaluation of osteoarthritis severity according to a previous study (79).

### Identification of drug target.

To identify key factors driving FB remodeling, we combined a causal-inspired approach (P-NET) with a correlation-based approach (Seurat). This enabled us to identify high-confidence candidate targets that are both statistically correlated and biologically interpretable.

The P-NET algorithm integrates prior biological knowledge by embedding known gene-to-pathway relationships, enabling both prediction and interpretability of the deep learning model (51). We used the gene expression profiles of FBs from different groups as input features and aggregated genes into corresponding pathway nodes according to Reactome annotations. These pathway nodes constituted a sparse feedforward intermediate layer. The model was trained using cross-validation and L1/L2 regularization to prevent overfitting and enhance generalizability. For model interpretability, we applied DeepLIFT (Deep Learning Important FeaTures) to compute feature importance scores, quantifying each gene’s contribution to the model’s predictions. Scores from different cross-validation folds were aggregated to generate a final gene-level ranking. Highly ranked genes were defined as P-NET–selected features, representing upstream regulatory nodes that may have causal significance at the pathway level.

In parallel, we employed Seurat to perform differential expression analysis of FBs between different groups. This approach emphasizes the statistical significance of expression changes and reveals transcriptional features associated with FB remodeling from a data-driven perspective.

Next, the top 150 upregulated and downregulated DEGs of FBs between different groups were submitted to the Connectivity Map for drug-response prediction, and complementary drug-to-gene interaction information was retrieved from DGIdb to construct a refined, data-driven drug database. By intersecting P-NET–identified genes and Seurat-derived DEGs with target genes included in this database, we obtained a set of candidate target genes and corresponding small-molecule compounds potentially capable of modulating FB function.

### Isolation of primary porcine FBs, MCs, and cell culture.

TMJ RTs were separated from 5-month-old female mini pigs under aseptic conditions and rinsed with PBS (Servicebio) to remove blood and other impurities. Tissue samples were then cut into approximately 1 mm^3^ sections and digested in 4 mg/mL Pronase for 1 hour, followed by digesting in 2 mg/mL collagenase P in FBS-free DMEM for 2 hours at 37°C. An equivalent volume of medium containing 10% FBS (Gibco) was added to terminate the reaction. Cells were pelleted by centrifugation at 300 g for 5 minutes. After removing the supernatant, cell pellet was resuspended with DMEM containing 10% FBS and 1% penicillin/streptomycin (Cienry).

Primary FBs displayed robust adhesive properties and replicated relatively quickly. For enrichment of primary FBs, we cultured cell suspension for 4 hours, and adherent cells were collected and then passaged for several times to enrich FBs (80). Additionally, unattached fractions of the cell suspension after the first 4 hours of culture were transferred into new dishes to selectively enrich for MCs. MCs, especially pericytes, can proliferate in media with lower concentrations of glucose and FBS (81, 82). Therefore, the growth of ECs and FBs could be effectively inhibited in this nutrient media. After stable adhesion of cells, the complete medium was replaced by porcine pericyte complete medium (Procell).

Cells were cultured in a humidified incubator at 37°C and 5% CO_2_. The medium was completely changed every 3–4 days. When the cultures reached 70%–80% confluence, the cells were subcultured using 0.25% trypsin-EDTA (Cienry). Cells at passage 3 were used in the following experiments. MCs were treated with TGF-β3 (PeproTech). FBs were treated with batimastat (MCE), sunitinib (MCE), or zaprinast (MCE).

### Chondrogenic induction.

Chondrogenic induction media was composed of DMEM supplemented with 10^−7^ mol dexamethasone (Sigma-Aldrich); 1 mM sodium pyruvate (Sigma-Aldrich); 50 μg/mL L-ascorbic-2-phosphate (Sigma-Aldrich); 40 μg/mL L-proline (Sigma-Aldrich); 1% insulin, transferrin, selenium (ITS, Beyotime); and 10 ng/mL TGF-β3 (PeproTech).

Cells cultured in a 6-well cell culture plate (Nest) underwent chondrogenic induction for 3 days and 7 days for qRT-PCR and Western blot analysis, respectively. For pellet culture, cells were first centrifuged for 5 minutes at 300*g* to form a pellet. After 3 weeks of chondrogenic induction, pellets were fixed in 4% PFA and prepared for paraffin sections and were stained with Alcian blue (Beyotime).

### Immunofluorescence staining.

Paraffin sections were deparaffined, and then antigen retrieval was performed using 0.1% trypsin-EDTA for 30 minutes at 37°C. Subsequently, sections were blocked with PBST (PBS + 0.1% Tween 20) supplied with 10% goat serum for 1 hour at 37°C, and then incubated with primary antibodies (ACTA2, 1:200, 67735-1-Ig, Proteintech; DCN, 1:50, PSH12-99, HuaBio; Aggrecan, 1:100, A11691, Abclonal; collagen type II, 1:100, 28459-1-AP, Proteintech; SOX9, 1:100, ab185966, Abcam; FGF2 1:100, BD-PT5549, Biodragon; BMP5 1:100, PK10384, Abmart; Ki67 1:100, 27309-1-AP, Proteintech; COL1A1 1:200, HA722517, HuaBio; APOE, 1:100, 66830-1-Ig, Proteintech) overnight at 4°C. Sections were incubated with the secondary antibodies for 1 hour at 37°C (goat anti-mouse Alexa Fluor 488, HA1125; goat anti-rabbit Alexa Fluor 594, HA1122; 1:500, HuaBio). Slices were mounted with Antifade Mounting Medium with DAPI (Solarbio) and detected by laser scanning confocal microscopy (Pannoramic 250 Flash, 3DHISTECH). Three areas were randomly selected to calculate percentages of Aggrecan^+^ area, collagen type II^+^ area, and SOX9^+^ cells using ImageJ (NIH) software (v1.54).

Primary porcine FBs were collected and resuspended in 35 mm confocal dishes (Nest) and incubated for 2 days. Cells were fixed in 4% PFA in PBS (pH 7.4, Servicebio) for 15 minutes at room temperature. After washing with ice-cold PBS 3 times, cells were then permeabilized with 0.1% Triton X-100 (Beyotime) for 10 minutes. After washing, cells were incubated with 3% BSA in PBST for 1 hour at 37°C to block unspecific binding of the antibodies. After decanting the solution, cells were further incubated with diluted primary antibody (APOE, 1:100, 66830-1-Ig, Proteintech; COL1A1, 1:100, HA722517, HuaBio; Smad4, 1:100, ET1604-12, HuaBio; ACTA2, 1:200, 67735-1-Ig, Proteintech) in 3% BSA in PBST overnight at 4°C, then incubated with diluted secondary antibody (goat anti-mouse Alexa Fluor 488, HA1125; goat anti-rabbit Alexa Fluor 594, HA1122; 1:500, HuaBio) in 3% BSA in PBST for 1 hour at 37°C. Cells were stained with DAPI (Solarbio) for 5 minutes at room temperature and washed with PBS for 3 times, then observed under a Zeiss LSM 980 microscope.

### RNA isolation and qRT-PCR.

The total RNA of FBs was purified with RNAeasy Animal RNA Isolation kit with Spin Column (Beyotime). RNA quantity and purity were determined using Nanodrop OneC (Thermo Fisher Scientific). RNA samples (260/280 ≥ 1.8) were used to obtain cDNA with HiScript III RT SuperMix for qPCR (Vazyme). qRT-PCR was performed using ChamQ Universal SYBR qPCR Master Mix (Vazyme). Primers for RT-qPCR are listed in Supplemental Table 1. Each sample was run in triplicate to ensure quantitative accuracy. The results were calculated using the 2^−ΔΔCt^ method. Gene expression levels were normalized to the housekeeping gene *GAPDH*.

### Western blot.

Cells were collected and lysed in 1× protein loading buffer containing DTT and boiled at 95°C for 10 minutes. Proteins were resolved by SDS-PAGE and transferred to PVDF membranes. After blocking in 5% skim milk in Tris-buffered saline with 0.1% Tween 20 for 1 hour at room temperature, membranes were cut and incubated with diluted primary antibodies overnight at 4°C. The primary antibodies included FN1 (1:1,000, T59537, Abmart), COL1A1 (1:1,000, HA722517, HuaBio), DCN (1:1,000, HA723504, HuaBio), ACTA2 (1:1,000, 67735-1-Ig, Proteintech), FGF2 (1:1,000, BD-PT5549, Biodragon), BMP5 (1:1,000, PK10384, Abmart), aggrecan (1:1,000, A11691, Abclonal), collagen type II (1:1,000, 28459-1-AP, Proteintech), SOX9 (1:1,000, ab185966, Abcam, UK), phospho-Smad1/5/9 (1:1,000, HA722566, HuaBio), Smad1/5/9 (1:1,000, ER64980, HuaBio), phospho-ERK1/2 (1:1,000, 28733-1-ap, Proteintech), ERK1/2 (1:5,000, 51068-1-ap, Proteintech), and GAPDH (1:10,000, 60004-1-Ig, Proteintech). Then, membranes were incubated with diluted HRP-conjugated goat anti-rabbit or anti-mouse secondary antibodies (1:10,000, HA1001, HA1006, HuaBio) for 1 hour at room temperature. Finally, enhanced chemiluminescence detection reagents (Fdbio Science) were used to scan membranes in the imaging system (Bio-Rad ChemiDoc MP).

### Micro-CT scanning, reconstruction, and microstructural analysis.

TMJ specimens were scanned with a high-resolution micro-CT system (SkyScan1276, Bruker). Scanning was performed at a source voltage of 60 kV and a current of 200 μA, with a 0.5 mm aluminum filter and voxel size of 13 μm. Images were acquired with 2 × 2 camera binning, with exposure time of 229 ms. Scanning was performed in step-and-shoot mode with flat-field correction and random movement disabled. The raw projection images were reconstructed using NRecon (v2.2.0.6). A region of interest (ROI) was defined within the trabecular bone area, excluding the cortical shell. The following standard bone morphometric parameters were calculated using CTAn software (Bruker) according to the guidelines (83): bone volume/total volume (%), trabecular thickness (μm), trabecular spacing (μm), and trabecular number (1/mm). All morphometric parameters were calculated using a 3D model–independent method based on distance transformation algorithms as implemented in CTAn. Three-dimensional visualization of the bone structure was performed using CTVol software (Bruker).

### Statistics.

All statistical analyses were performed with R (v4.3) and GraphPad Prism (v9.0). Data are presented as mean ± SD. Comparisons between 2 groups were performed using unpaired, 2-tailed Student’s *t* test. For multiple group comparisons, 1-way ANOVA followed by Tukey’s honestly significant difference post hoc test or Kruskal-Wallis test followed by Dunn’s multiple-comparison test were used. A *P* value less than 0.05 was considered statistically significant.

### Study approval.

The study protocols were reviewed and approved by the Animal Ethics Committee of Zhejiang Chinese Medical University, China (approval 20230227-08). All procedures were conducted in strict accordance with the *Guide for the Care and Use of Laboratory Animals* (National Academies Press, 2011).

### Data availability.

All data supporting the findings of this study are included in the supplemental materials and Supporting Data Values file, or are available from the corresponding authors upon reasonable request. Sequencing data have been deposited in the Genome Sequence Archive in National Genomics Data Center, China National Center for Bioinformation (accession CRA034150).

## Author contributions

WY designed the study, performed animal and cell experiments, acquired data, analyzed data, and drafted and revised the manuscript. YC designed the study, performed animal and cell experiments, analyzed data, and drafted and revised the manuscript. RY performed bioinformatics analysis, analyzed data, supported the interpretation of results, and revised the manuscript. WL performed animal experiments and acquired data. CW analyzed data and assisted in animal model validation. YW and QD participated in data collection. WL and MZ critically reviewed the manuscript. XC supervised the study and critically reviewed the manuscript. JS supervised the study, provided reagents for animal and cell experiments, and critically reviewed the manuscript. The order of co-first authors was determined based on their overall contributions to the study and was approved by all authors.

## Conflict of interest

The authors have declared that no conflict of interest exists.

## Funding support

The following organizations provided funding support:

- National Natural Science Foundation of China (grant 82170984).
- Joint TCM Science & Technology Projects of National Demonstration Zones for Comprehensive TCM Reform (grant GZY-KJS-ZJ-2025-094).
- Scientific Research Fund of Zhejiang University (grant XY2024036).

## Supplementary Material

Supplemental data

Unedited blot and gel images

Supporting data values

## Version 1 02/10/2026

In-Press Preview

## Version 2 07/08/2026

Electronic publication
