# Supplementary material for: Distinct mural cells and fibroblasts drive fibrochondrogenesis in retrodiscal tissue following temporomandibular joint disc displacement
Source: JCI Insight. 2026 Feb 10;11(13):e196343. doi: 10.1172/jci.insight.196343 (PMC13460816; doi:10.1172/jci.insight.196343)
Supplement: Supplementary file 1 [file jciinsight-11-196343-s103.pdf]

Supplemental Figures

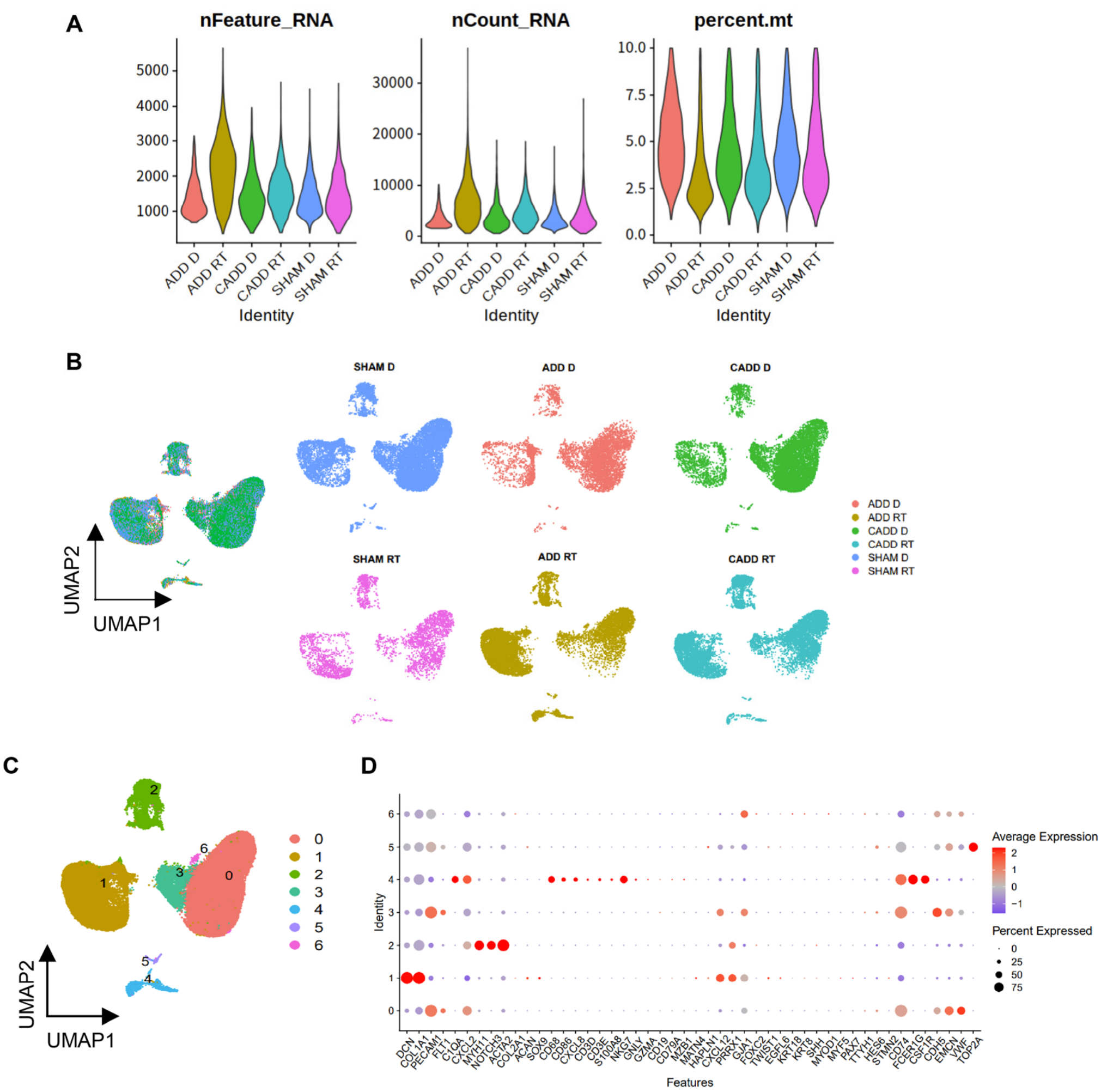

Supplemental Figure 1. Quality control, batch effect control, cell clustering and annotation of scRNA-seq data. (**A**) Analysis of the number of genes, UMI counts and percentage of mitochondrial genes. (**B**) Cell distributions in different groups after batch effect correction are shown in UMAP plots. (**C**) Unsupervised clustering identifies seven distinct cell clusters. (**D**) Dot Plot of marker genes from published studies used for cell cluster annotation.

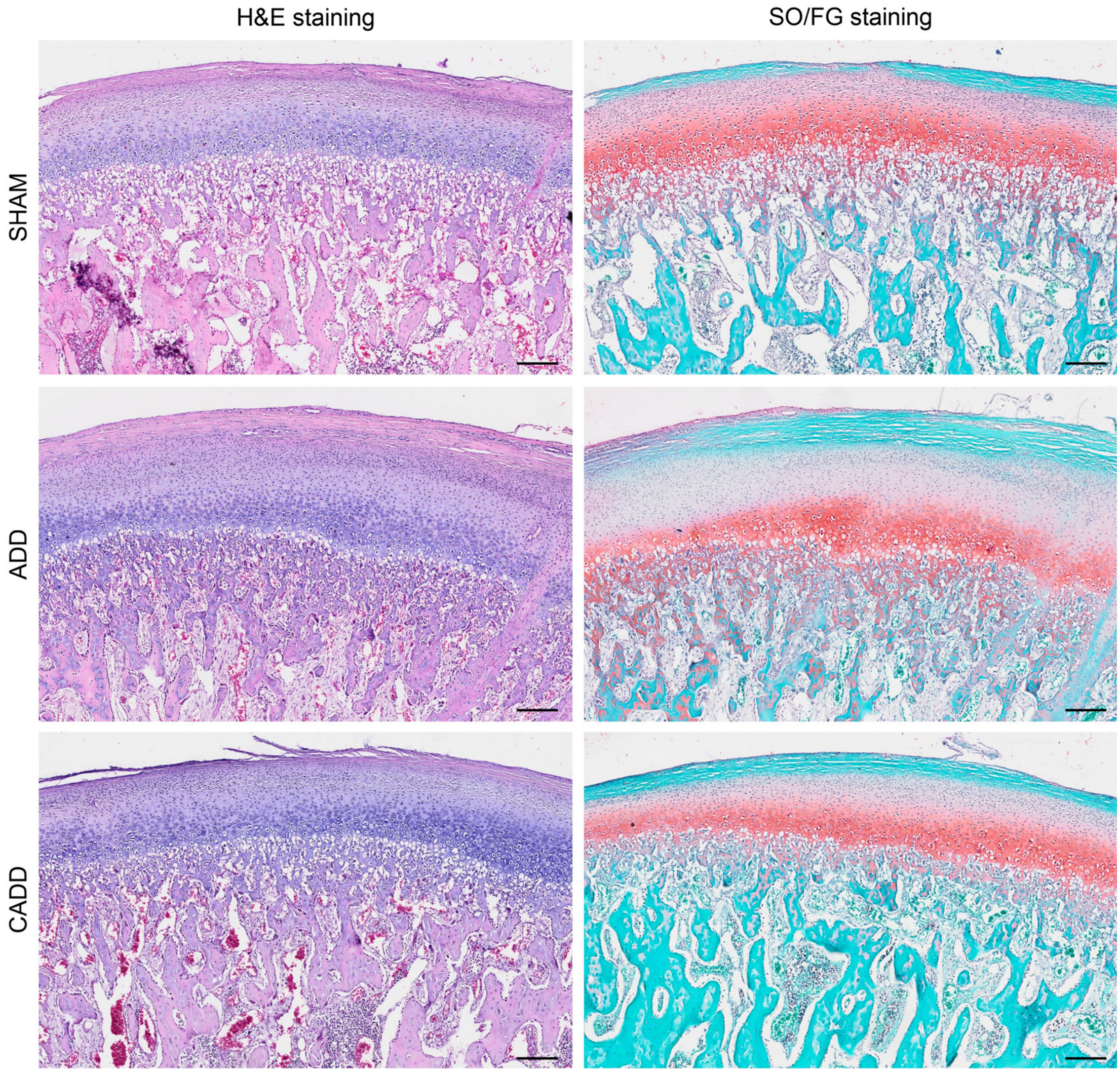

Supplemental Figure 2. Histological evaluation of porcine condyle after ADD induction. Hematoxylin and Eosin (H&E) staining (left panels) and Safranin-O and Fast Green (SO/FG) staining (right panels) of condyles from SHAM, ADD and CADD groups 5 weeks after surgery. Scale bars: 200 µm.

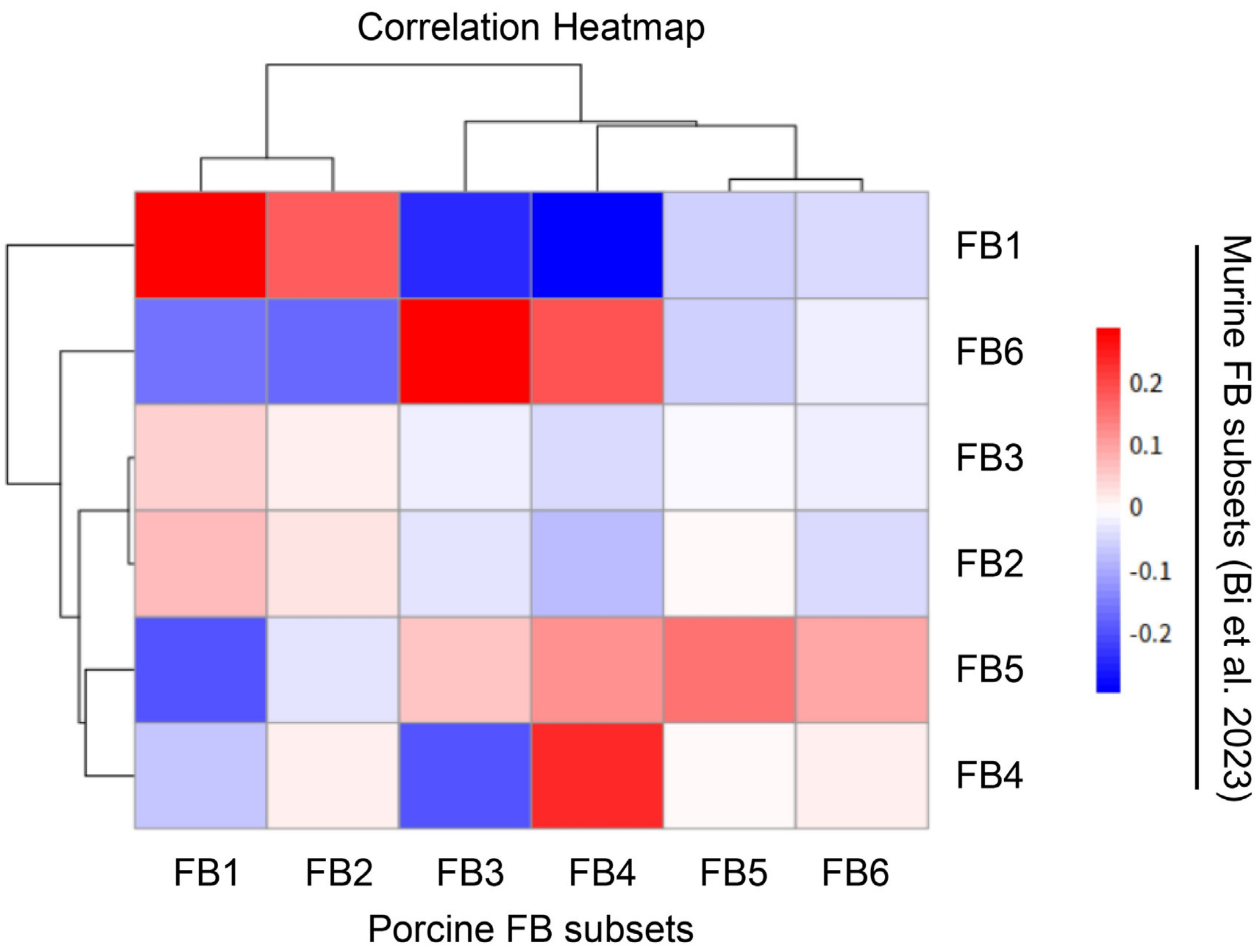

Supplemental Figure 3. Correlation of transcriptomic profiles between porcine and murine TMJ fibroblast subsets. The heatmap shows cross-species correlation between porcine fibroblast clusters and previously defined murine fibroblast subsets (Bi et al., 2023). Murine FB1 is defined as fibrillogenesis and fibrillary homeostasis related FBs; FB2, as proliferation related FBs; FB3, as apoptosis related non-chondrogenic FBs; FB4, as chondrogenesis inhibition related FBs; FB5 and FB6, as chondrogenic FBs.

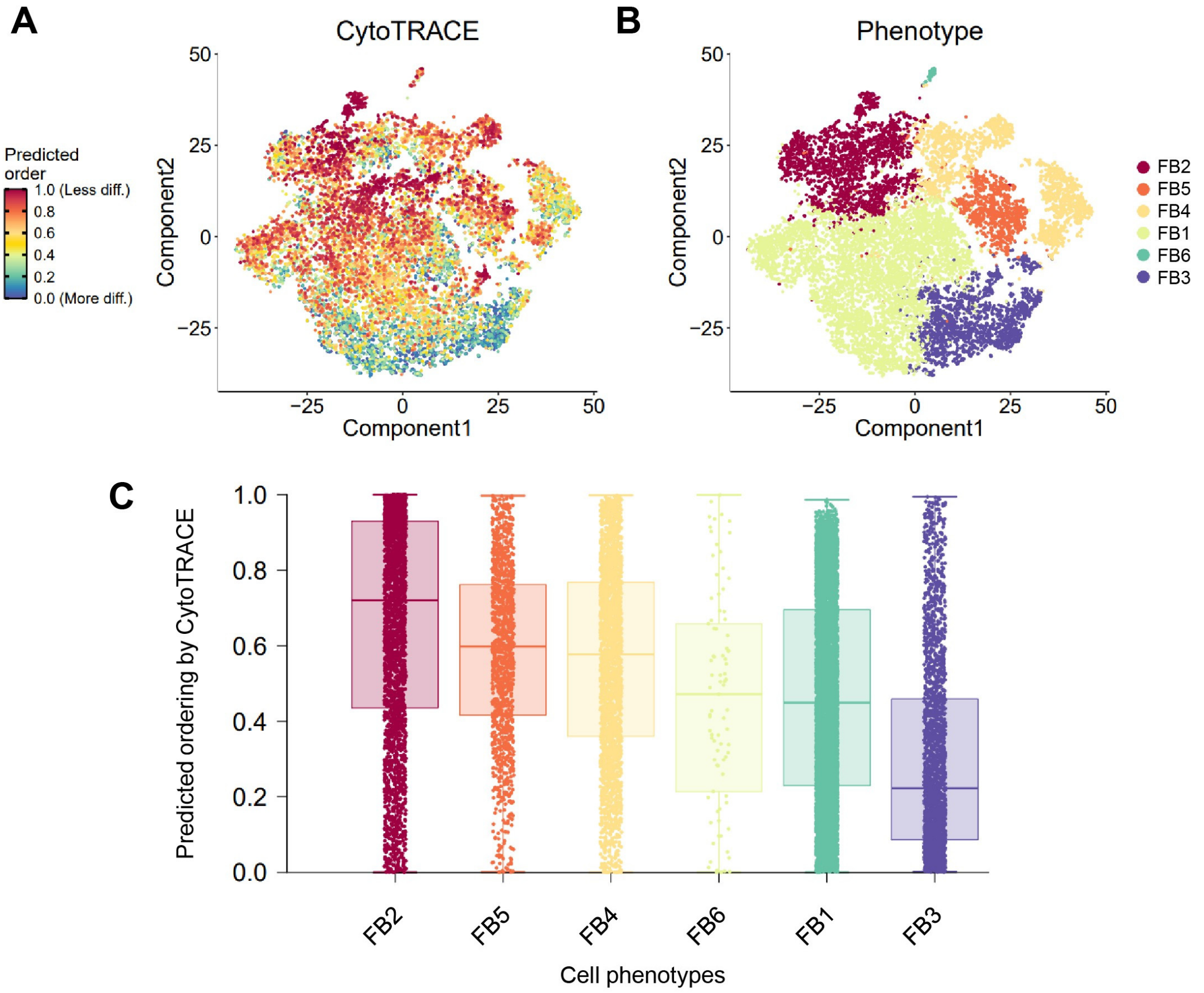

Supplemental Figure 4. CytoTRACE analysis of fibroblast subclusters. (**A**) Fibroblasts colored by developmental potential order. (**B**) Fibroblasts colored by different phenotypes. (**C**) Boxplot showing developmental potential order of fibroblast subclusters.

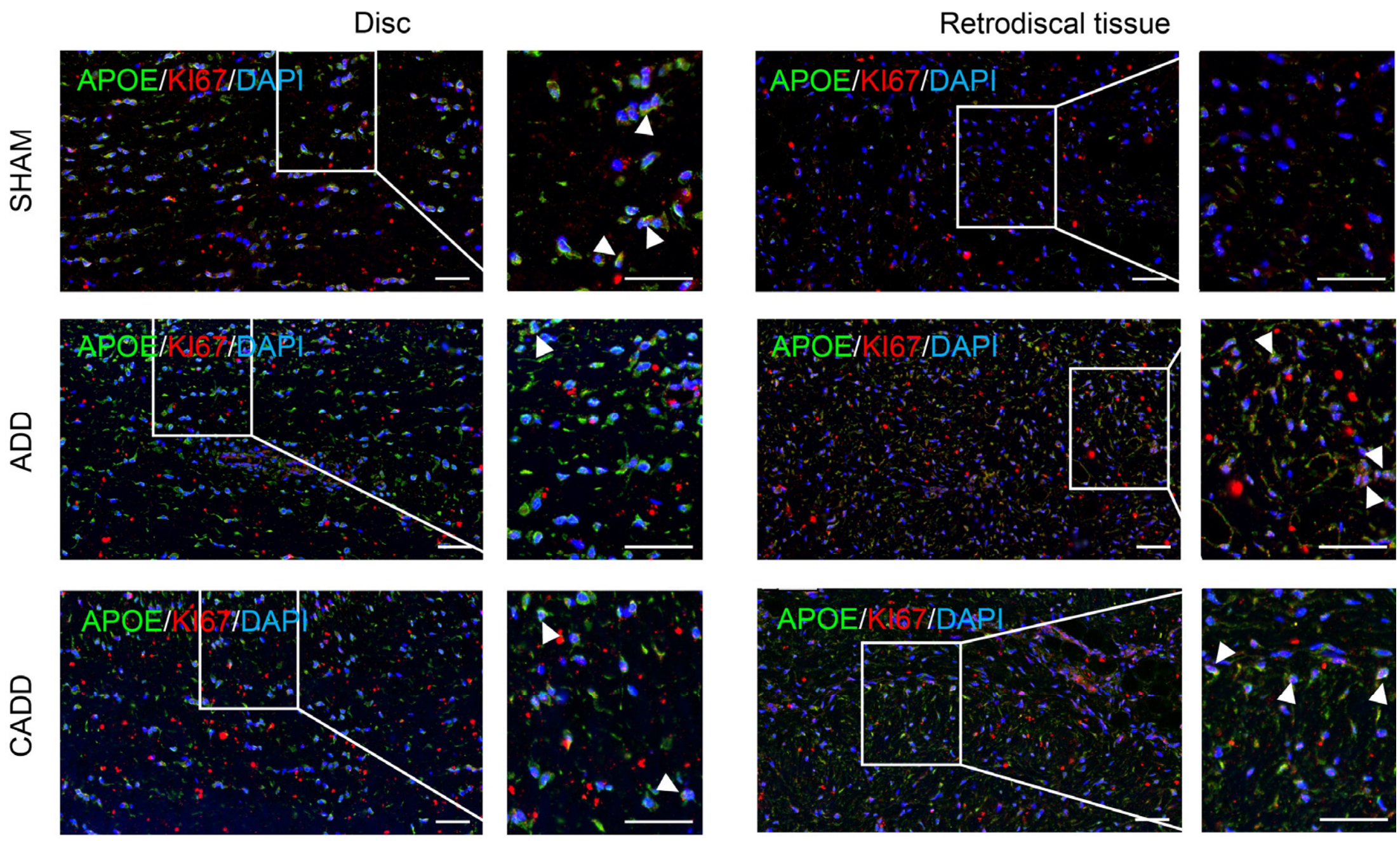

Supplemental Figure 5. Identification of proliferating FB2 subcluster. Representative images of TMJ discs and retrodiscal tissues subjected to immunofluorescence staining for KI67 and APOE in different groups. Green: APOE; Red: KI67; Blue: DAPI; White arrowhead: Cells co-expressing of APOE and KI67. Scale bars: 50 μm.

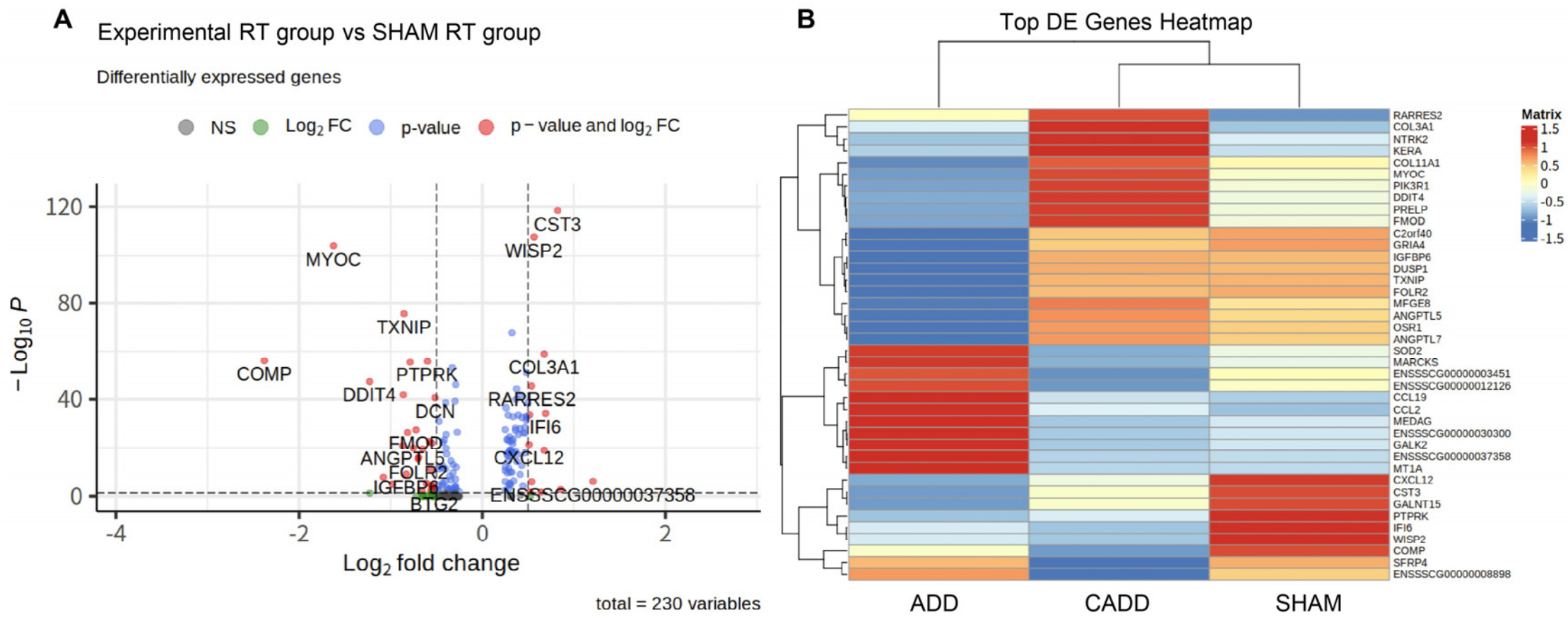

Supplemental Figure 6. Gene expression differences between experimental groups. (**A**) Volcano plot showing differentially expressed (DE) genes between Experimental (ADD+CADD) RT group and SHAM RT group. (**B**) Heatmap illustrating the expression patterns of DE genes across groups.

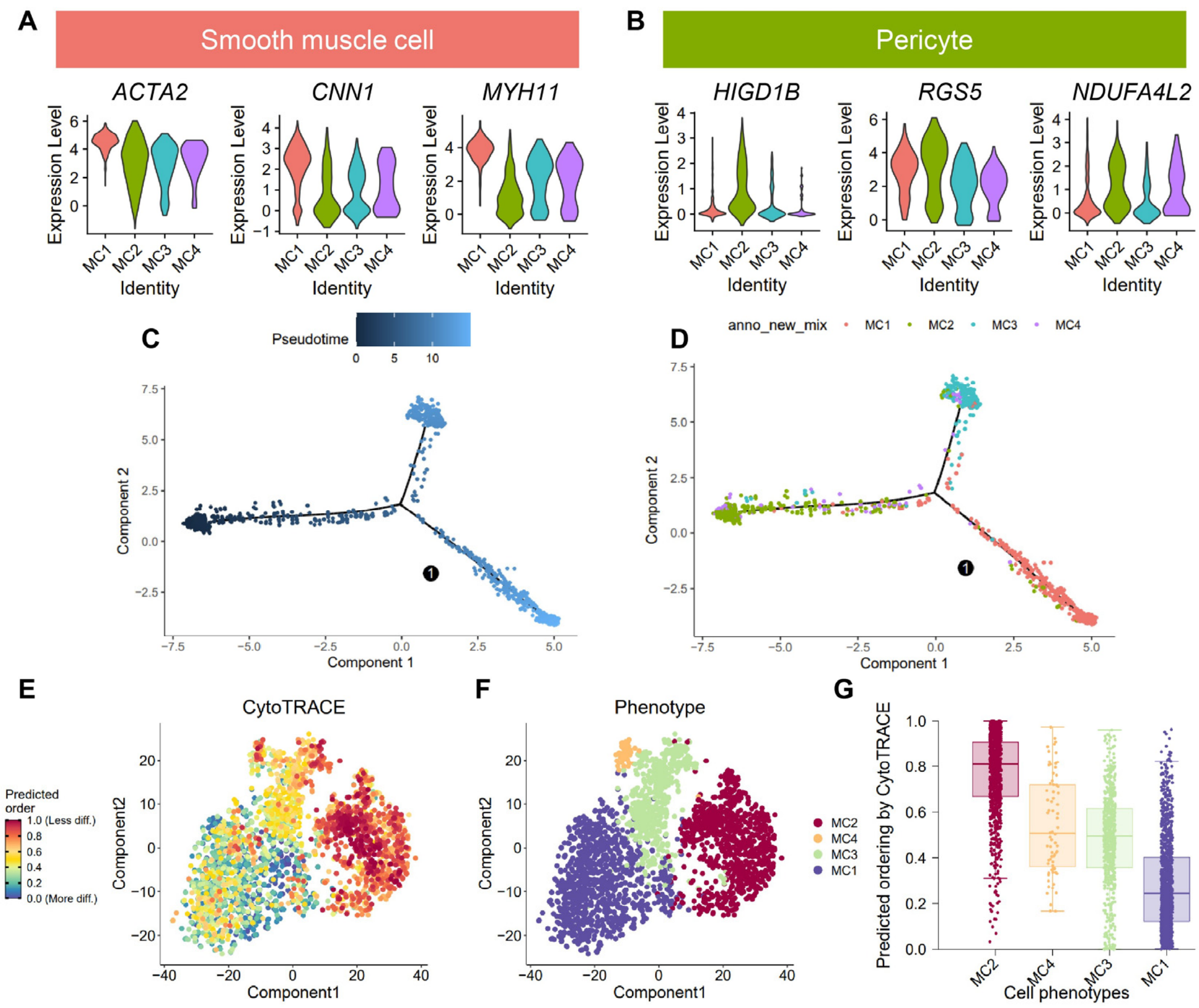

Supplemental Figure 7. Identification of mural cell subclusters and pseudotime trajectory analysis. (**A**) Violin plots showing the expression of smooth muscle cell marker genes in mural cell (MC) subclusters. (**B**) Violin plots showing the expression of pericyte marker genes in mural cell subclusters. (**C**) Monocle analysis showing the trajectory order of mural cells by pseudotime value. (**D**) Monocle analysis revealing two developmental branches of mural cells. (**E**) Mural cells colored by their developmental potential order. (**F**) Mural cells colored by different phenotypes. (**G**) Boxplot showing developmental potential order of mural cell subclusters.

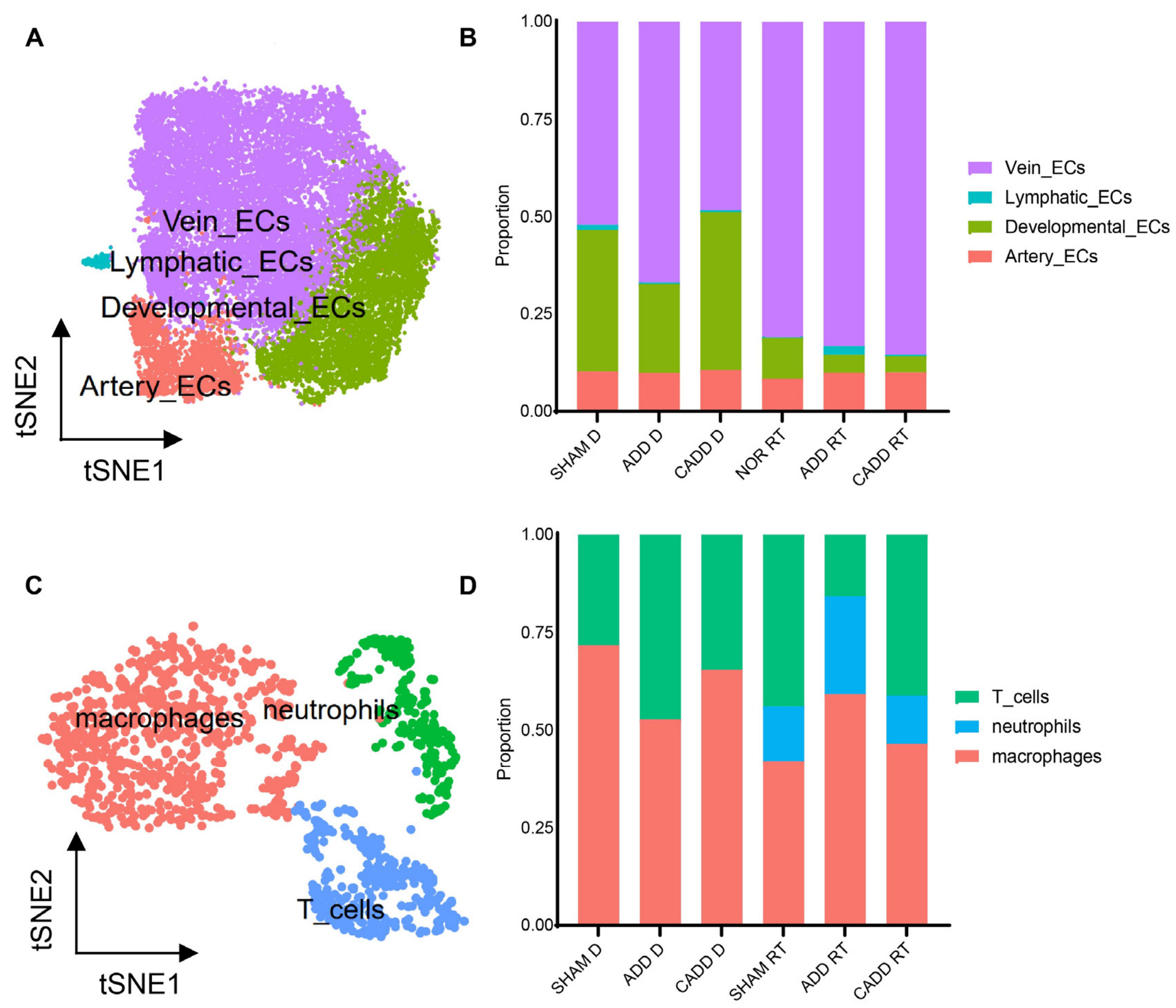

Supplemental Figure 8. Cellular feature changes in endothelial, immune and mural cells after ADD induction. (**A**) Four endothelial cell subclusters are identified, annotated according to previous studies and visualized by t-SNE plot. (**B**) Bar graph showing the fraction of endothelial cell subtypes by tissue origins and groups. (**C**) Three immune cell subclusters are identified, annotated according to previous studies and visualized by t-SNE plot. (**D**) Bar graph showing the fraction of immune cell subtypes by tissue origins and groups.

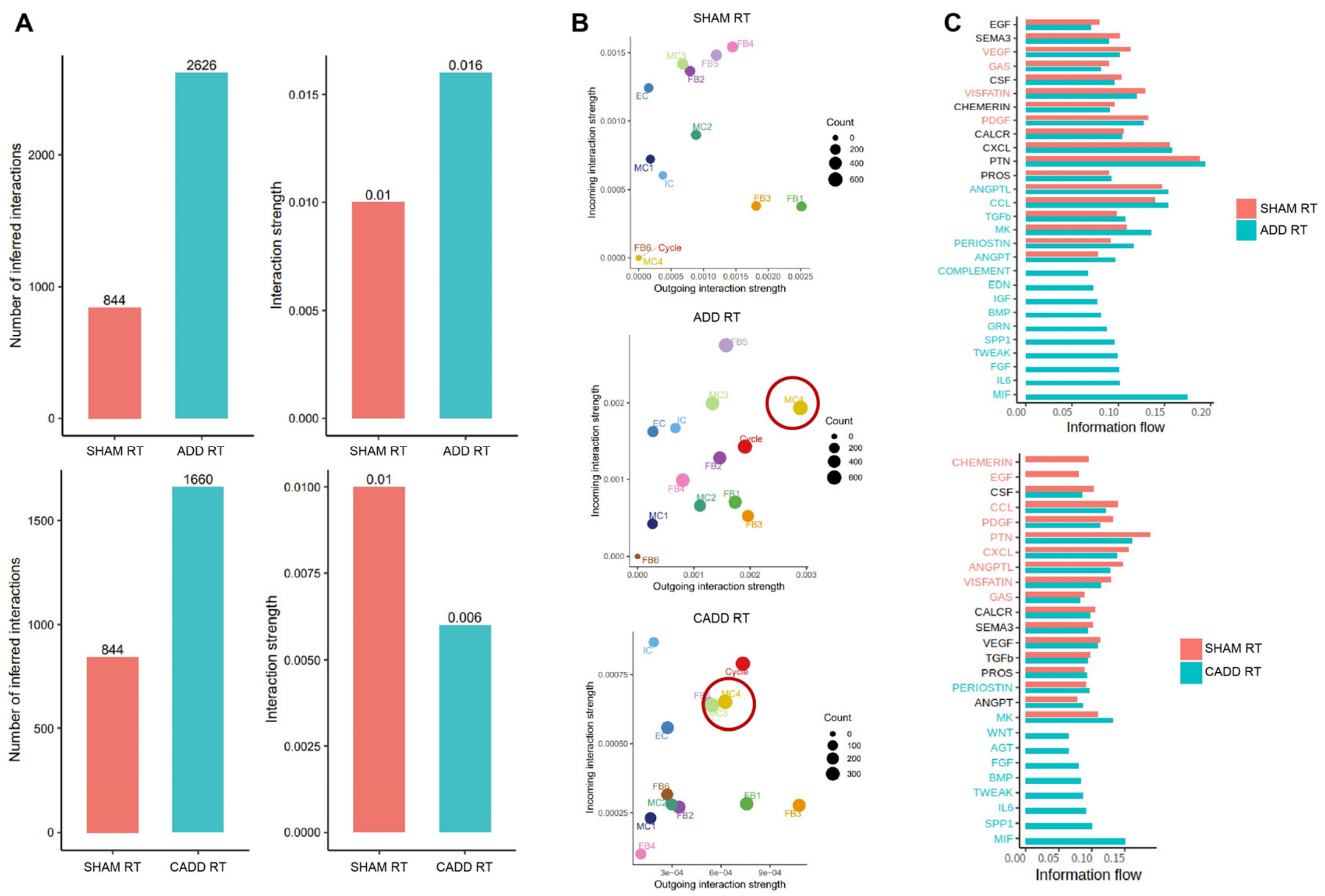

Supplemental Figure 9. Intercellular crosstalk regulating adaptive remodeling of the retrodiscal tissue. (**A**) Total number of possible interactions (left panel) and interaction strength (right panel) between SHAM and ADD RT groups, SHAM and CADD RT groups. (**B**) Incoming and outgoing interaction strengths of all cell types identifying prominent signaling sources and targets. (**C**) Significant signaling pathways are ranked based on differences in overall information flow, calculated by summarizing all communication probabilities in a given inferred network. Signaling colored red are more enriched in SHAM RT group and signaling color green are more enriched in ADD or CADD RT group.

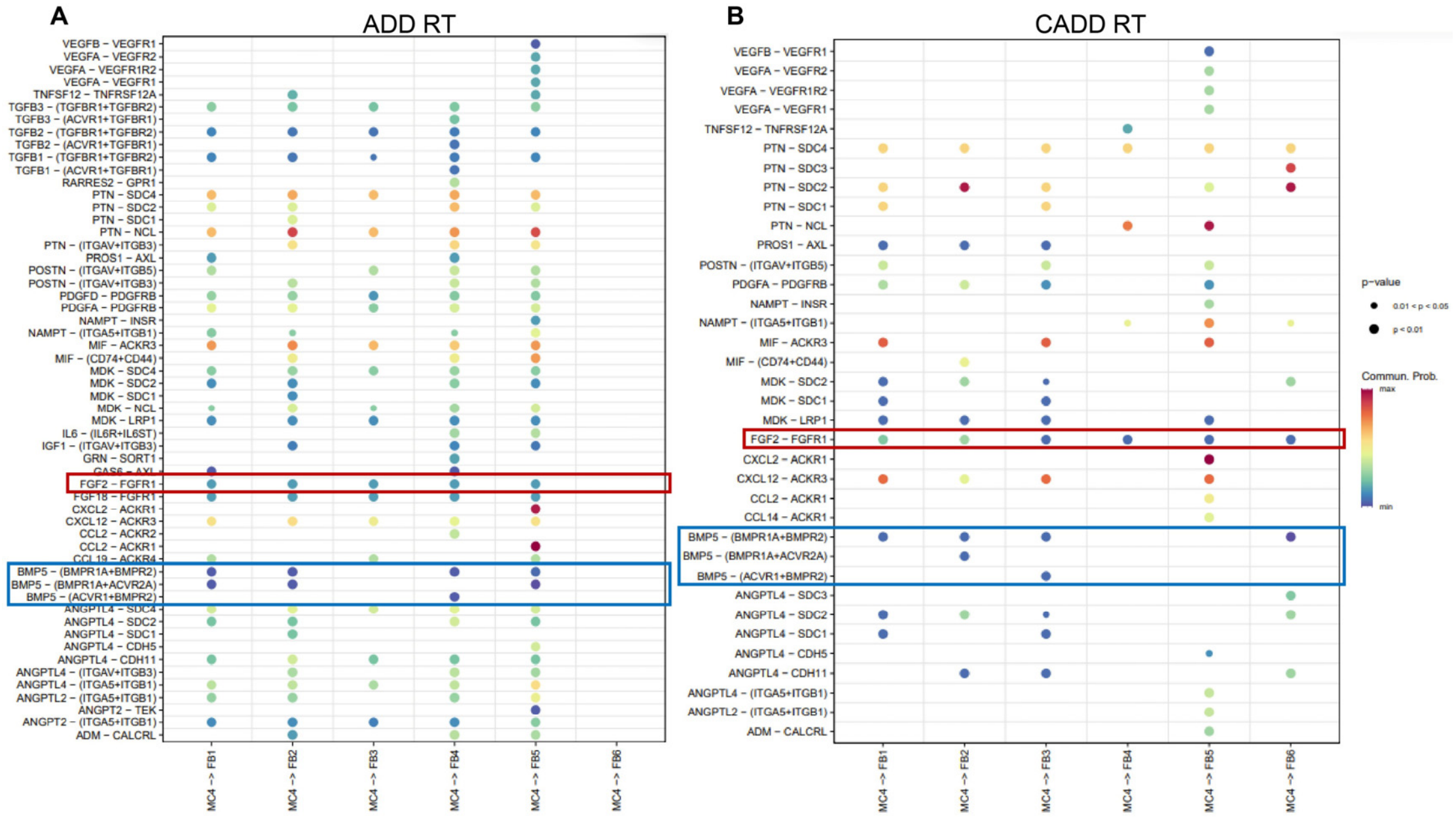

Supplemental Figure 10. Receptor-ligand signaling communication probabilities from MC4 to FB subtypes in ADD RT (A) and CADD RT (B).

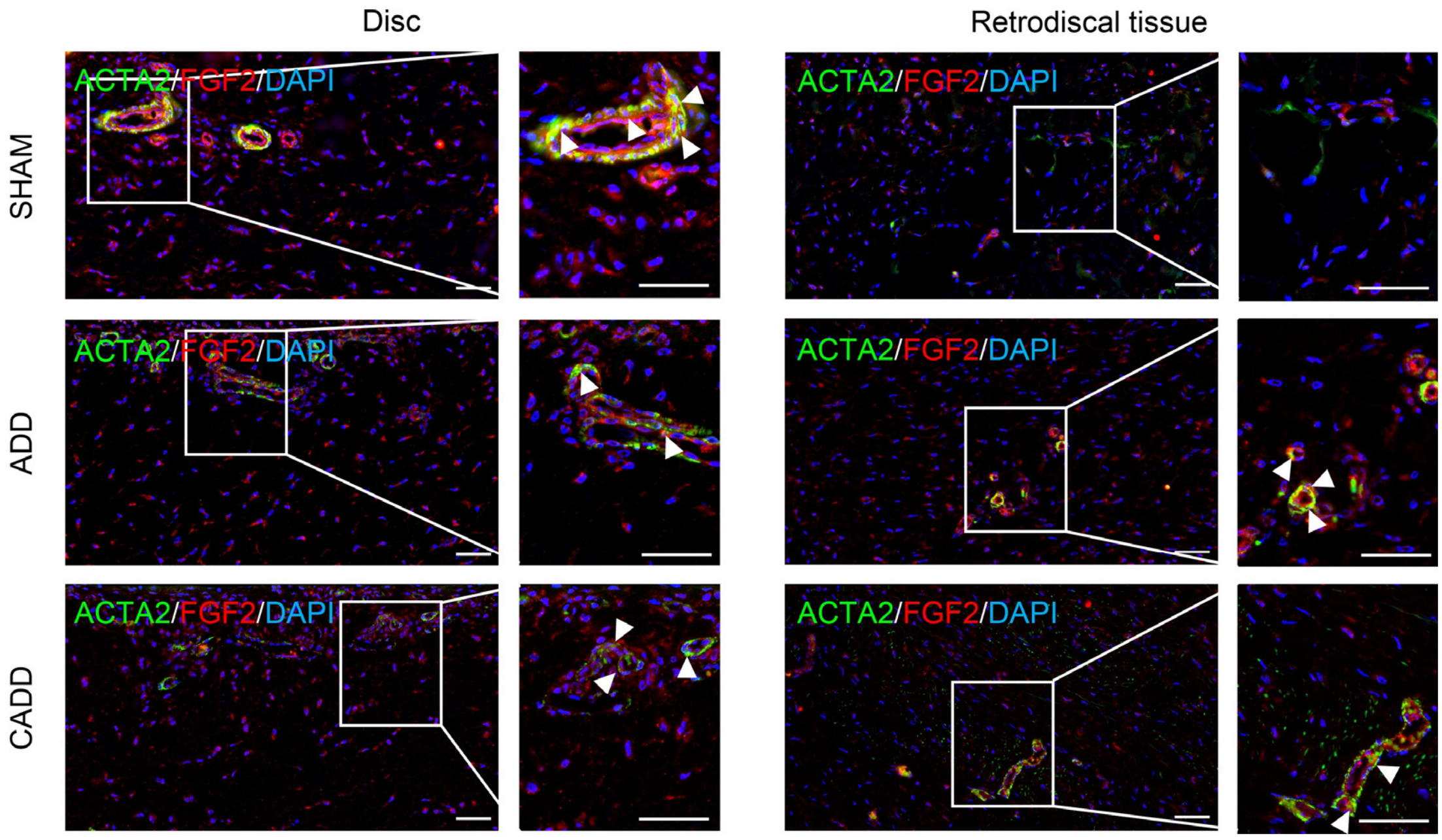

Supplemental Figure 11. Expression of FGF2 in mural cells. Representative images of TMJ discs and retrodiscal tissues subjected to immunofluorescence staining for ACTA2 and FGF2 in different groups. Green: ACTA2; Red: FGF2; Blue: DAPI; White arrowhead: Cells co-expressing of ACTA2 and FGF2. Scale bars: 50 μm.

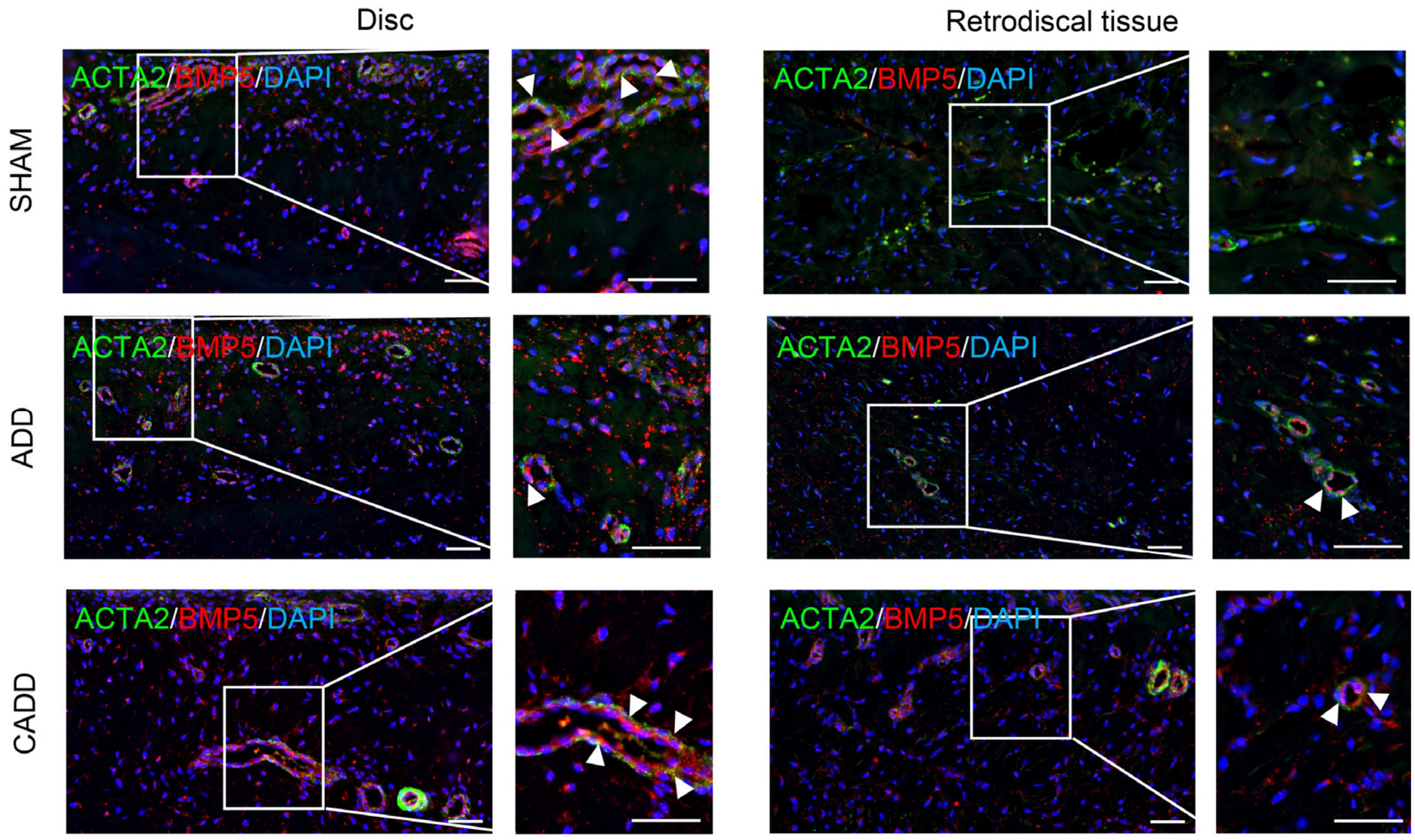

Supplemental Figure 12. Expression of BMP5 in mural cells. Representative images of TMJ discs and retrodiscal tissues subjected to immunofluorescence staining for ACTA2 and BMP5 in different groups. Green: ACTA2; Red: BMP5; Blue: DAPI; White arrowhead: Cells co-expressing of ACTA2 and BMP5. Scale bars: 50 μm.

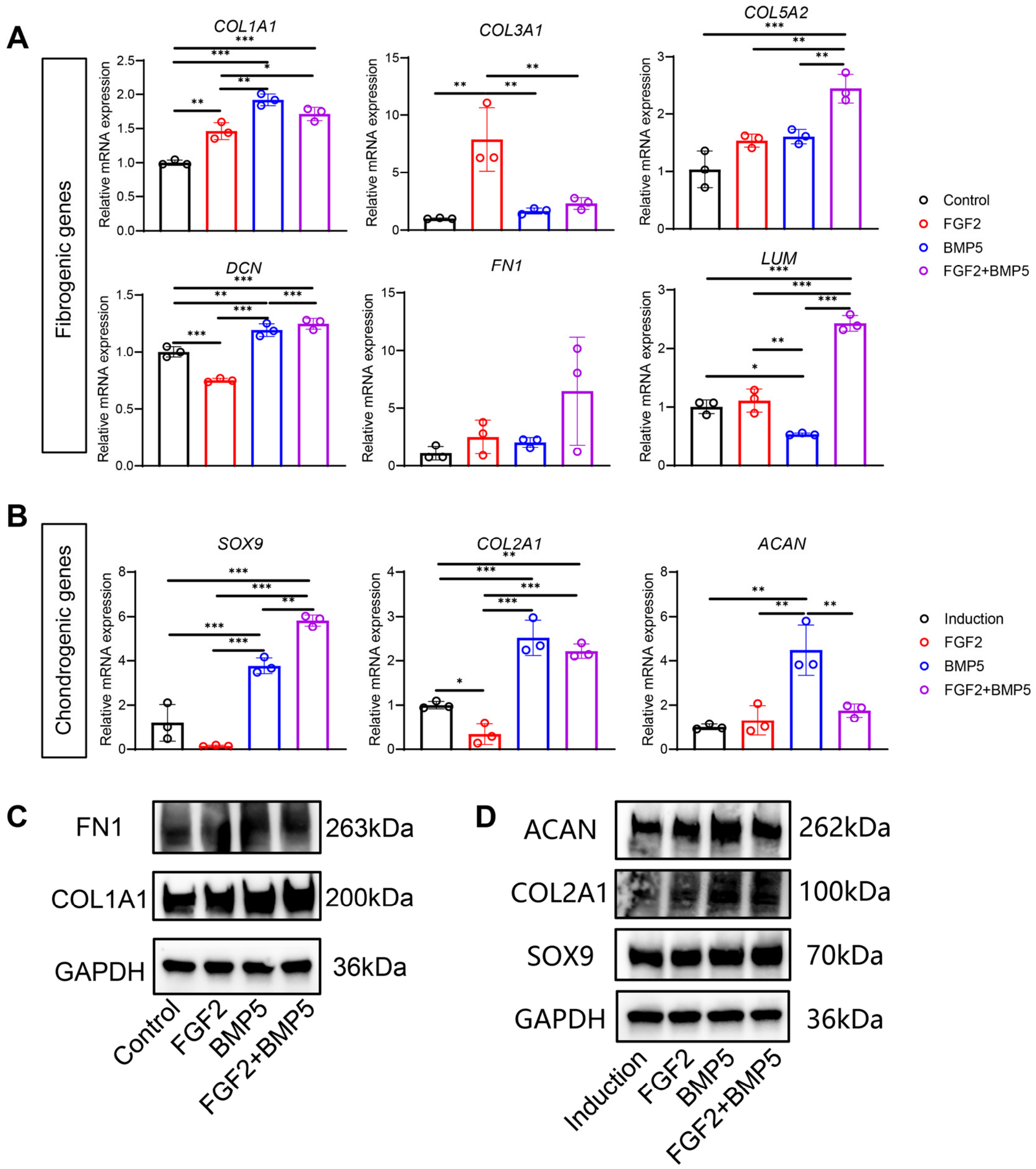

Supplemental Figure 13. FGF2 and BMP5 signaling enhance ECM synthesis and chondrogenic differentiation of fibroblasts. Evaluation of fibrogenic gene expressions (**A**) and protein levels (**C**) treated with FGF2, BMP5 and FGF2+BMP5. Evaluation of chondrogenic gene expressions (**B**) and protein levels (**D**) treated with FGF2, BMP5 and FGF2+BMP5 under chondrogenic induction. Fold changes were normalized with *GAPDH* (n=3). Statistical significance was determined by 1-way ANOVA. Data represent mean ± SD. *$P < 0.05$, **$P < 0.01$, ***$P < 0.001$. For representative western blot, 3 replicates were conducted.

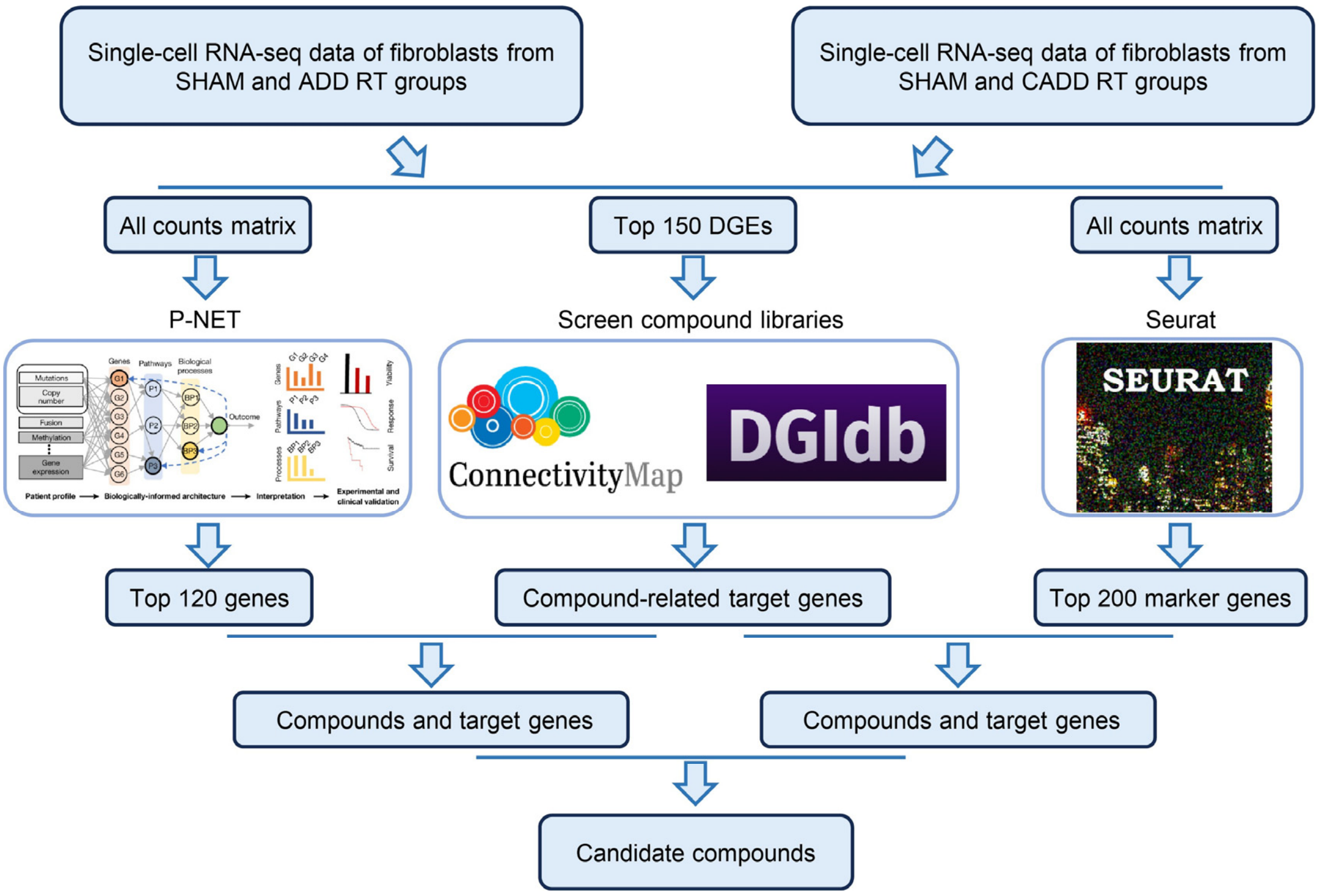

Supplemental Figure 14. Schematic workflow of candidate compound selection.

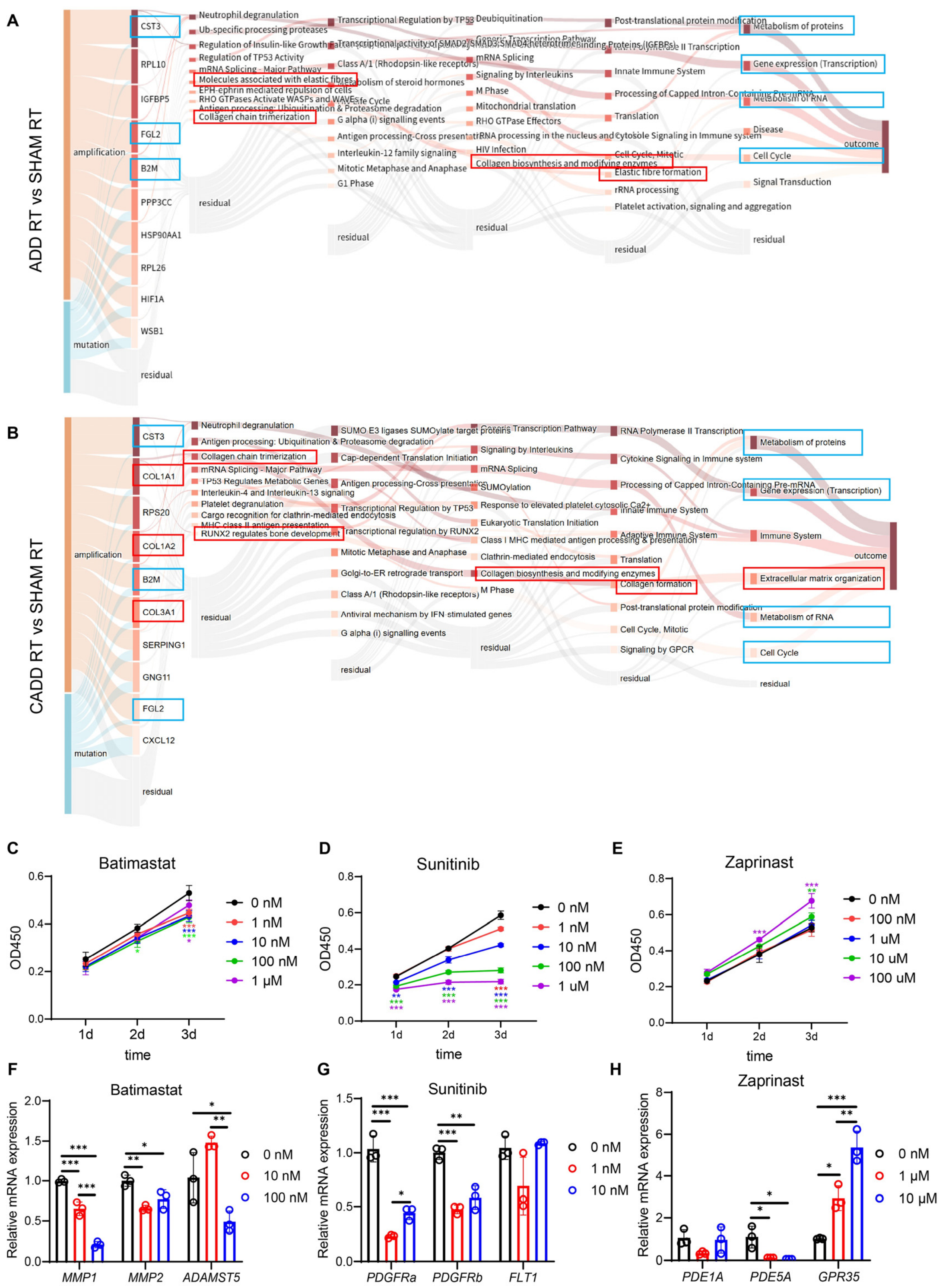

Supplemental Figure 15. Selection of potential candidate compounds enhancing remodeling capability of fibroblasts. Visualization of inner layers of P-NET shows the estimated relative importance of different nodes in each layer of fibroblasts from ADD RT (**A**) and CADD RT (**B**) groups using the Sankey diagram. Nodes on the far left represent feature types, the nodes in the

second layer represent genes, the next layers represent higher-level biological entities. Nodes with darker colors are more important, while transparent nodes represent the contribution of residual nodes in each layer. CCK8 assay were used to measure cytotoxicity of Batimastat (**C**), Sunitinib (**D**) and Zaprinast (**E**) under different concentrations (n=4). Statistical significance was determined by 2-way ANOVA. Data represent mean ± SD. *$P < 0.05$, **$P < 0.01$, ***$P < 0.001$. Data represent mean ± SD. *$P < 0.05$, **$P < 0.01$, ***$P < 0.001$. Evaluation of target gene expressions treated with Batimastat (**F**), Sunitinib (**G**) and Zaprinast (**H**) using qRT-PCR. Fold changes were normalized with *GAPDH* (n=3). Statistical significance was determined by 1-way ANOVA. Data represent mean ± SD. *$P < 0.05$, **$P < 0.01$, ***$P < 0.001$.

Supplemental Tables

Supplemental Table 1 Primer information

Gene symbol	Primer sequence (5’-3’)
COL1A1	Forward: CTGGTACGGCGAGAGCATGACC<br>Reverse: GGAGGAGCAGGGCCTTCTTGAG
COL3A1	Forward: GCTGCCATCGCTGGTGTTG<br>Reverse: TGAAGTCATAATCTCGTCGGTGTTG
COL5A2	Forward: TGGGGCTAGTGCTGAAAGAA<br>Reverse: CACCTCCAGTTGTATGCGGA
DCN	Forward: GATGCAGCTAGCCTGAAAGG<br>Reverse: TCACACCCGAATAAGAAGCC
FN1	Forward: ATATATCATTTCATGTCATCCCGTG<br>Reverse: CTTGTGCCTCTTCTGGTCTTTTAG
LUM	Forward: TCTGCTGGAGCTGGATCTCT<br>Reverse: CGCAAATGTTTGATCTTGGA
SOX9	Forward: GGGCTCTGTGCTCTACTCCA<br>Reverse: AGGTCTGGTGAGCTGTGTGT
COL2A1	Forward: ACTCCTGGCACGGATGGTC<br>Reverse: CTTTCTCACCAACATCGCCC
ACAN	Forward: CCCAACCAGCCTGACAACTT

	Reverse: CCTTCTCGTGCCAGATCATCA
APOE	Forward: GTGGGTTGCTTTGGTGGTAAC<br>Reverse: GCAGGTAATCCCAGAAGCGG
GAPDH	Forward: TCACGACCATGGAGAAGGCT<br>Reverse: CAGGAGGCATTGCTGATGATC

Supplemental Table 2 List of drugs and targets

Drug	Mechanism of action	Target
Batimastat	Matrix metalloprotease inhibitor	MMP1|ADAM28|ADAMTS5|MMP12|MMP16|MMP2|MMP8
Sunitinib	FLT3 inhibitor|KIT inhibitor|PDGFR inhibitor|RET inhibitor|VEGFR inhibitor	PDGFRB|FLT1|FLT3|FLT4|KDR|KIT|PDGFRA|RET
Zaprinast	Phosphodiesterase inhibitor	PDE5A|GPR35|PDE1A|PDE4D|PDE9A
Linifanib	PDGFR inhibitor|VEGFR inhibitor	CSF1R|KDR|PDGFRB|FLT1|FLT3|FLT4|PDGFRA|CSF1|KIT|RET|TEK
Sorafenib	RAF inhibitor|FLT3 inhibitor|KIT inhibitor|PDGFR inhibitor|RET inhibitor|VEGFR inhibitor	RET|BRAF|FLT3|KDR|RAF1|FLT1|FLT4|KIT|DDR2|PDGFRB|FGFR1|CYP2B6|CYP2C8|CYP3A5|PDGFB|SLCO1B3
