# Supplementary material for: Distinct mural cells and fibroblasts drive fibrochondrogenesis in retrodiscal tissue following temporomandibular joint disc displacement
Source: JCI Insight. 2026 Feb 10;11(13):e196343. doi: 10.1172/jci.insight.196343 (PMC13460816; doi:10.1172/jci.insight.196343)

Full unedited gel for Figure 4J

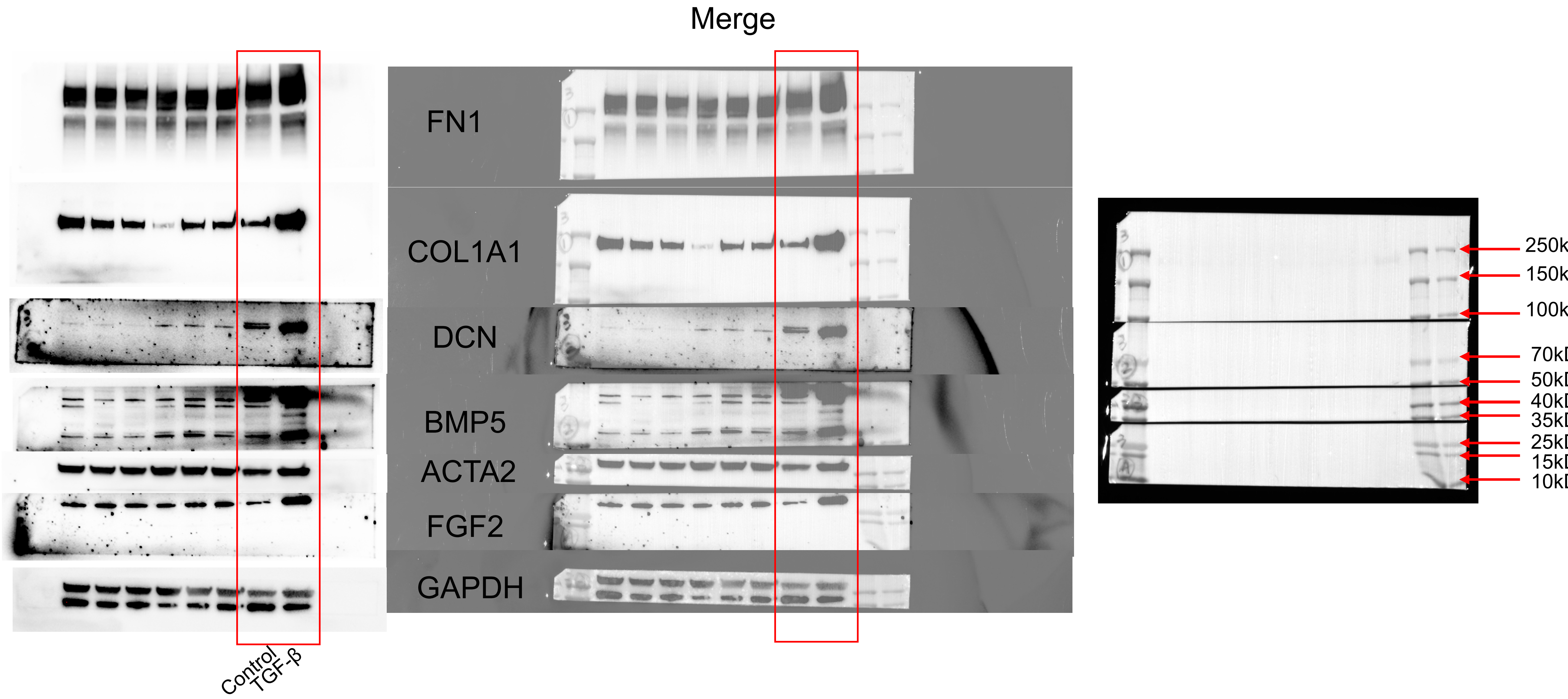

Full unedited gel for Figure 4K

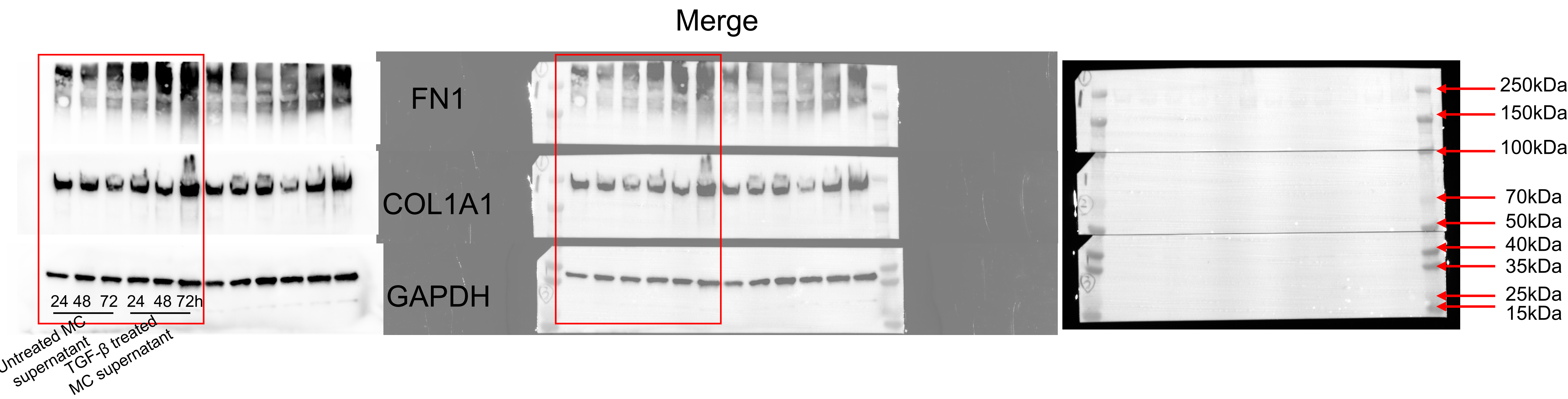

Full unedited gel for Figure 4L

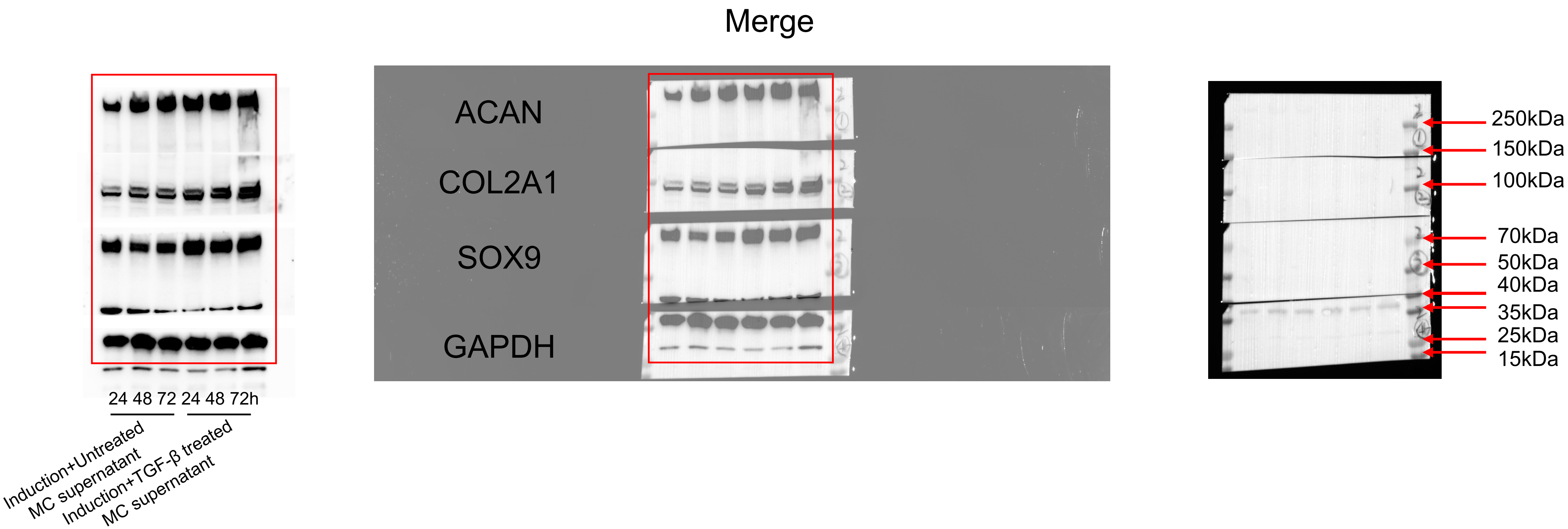

Full unedited gel for Figure 4M

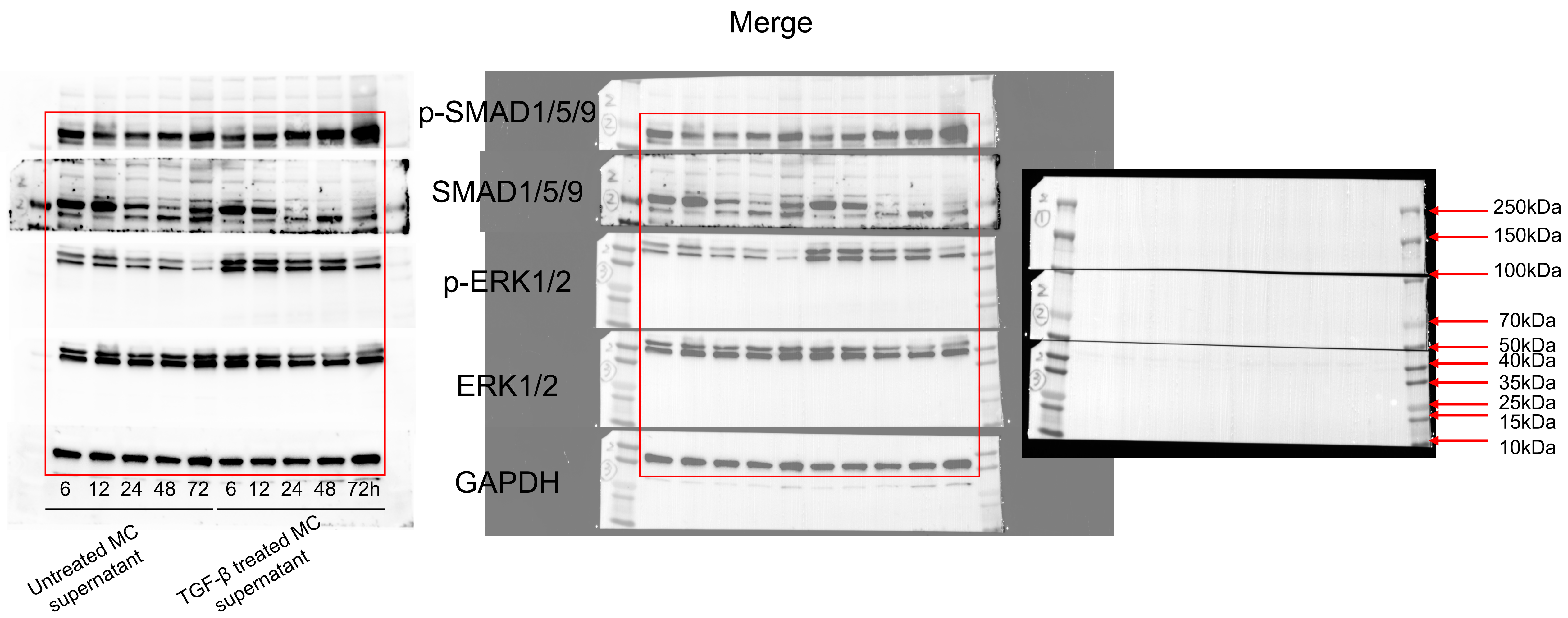

Full unedited gel for Figure 5B

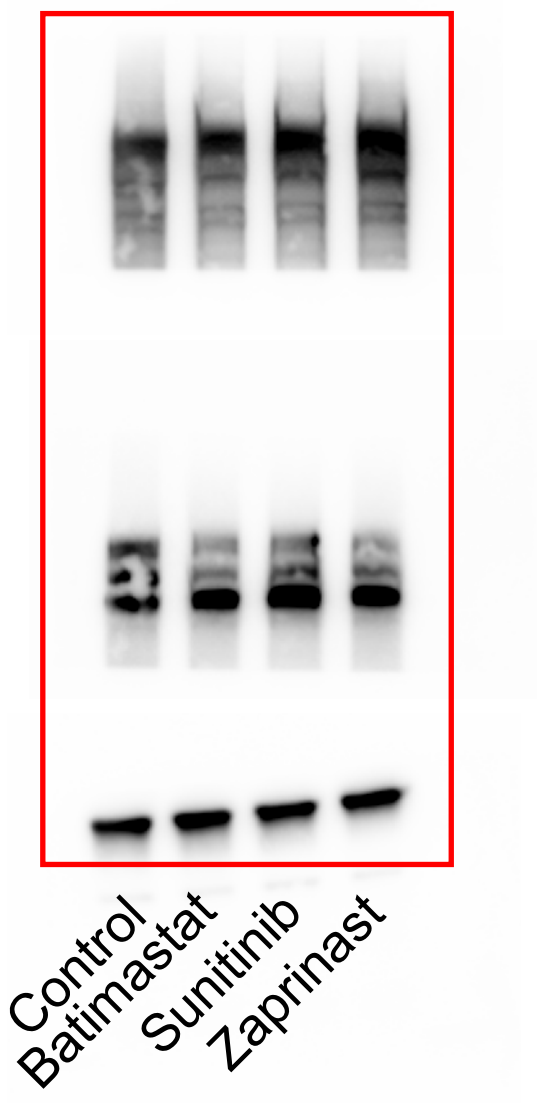

Merge

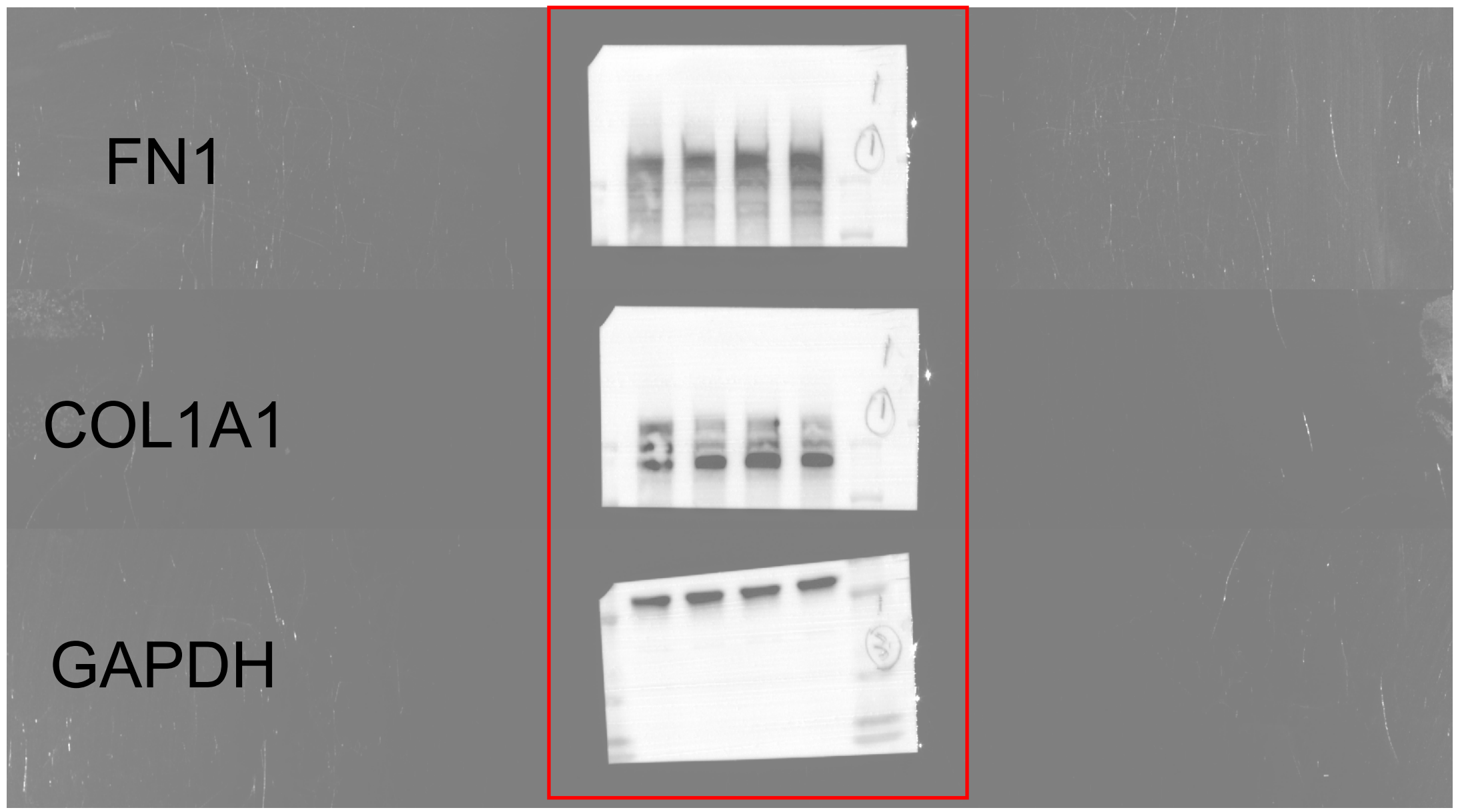

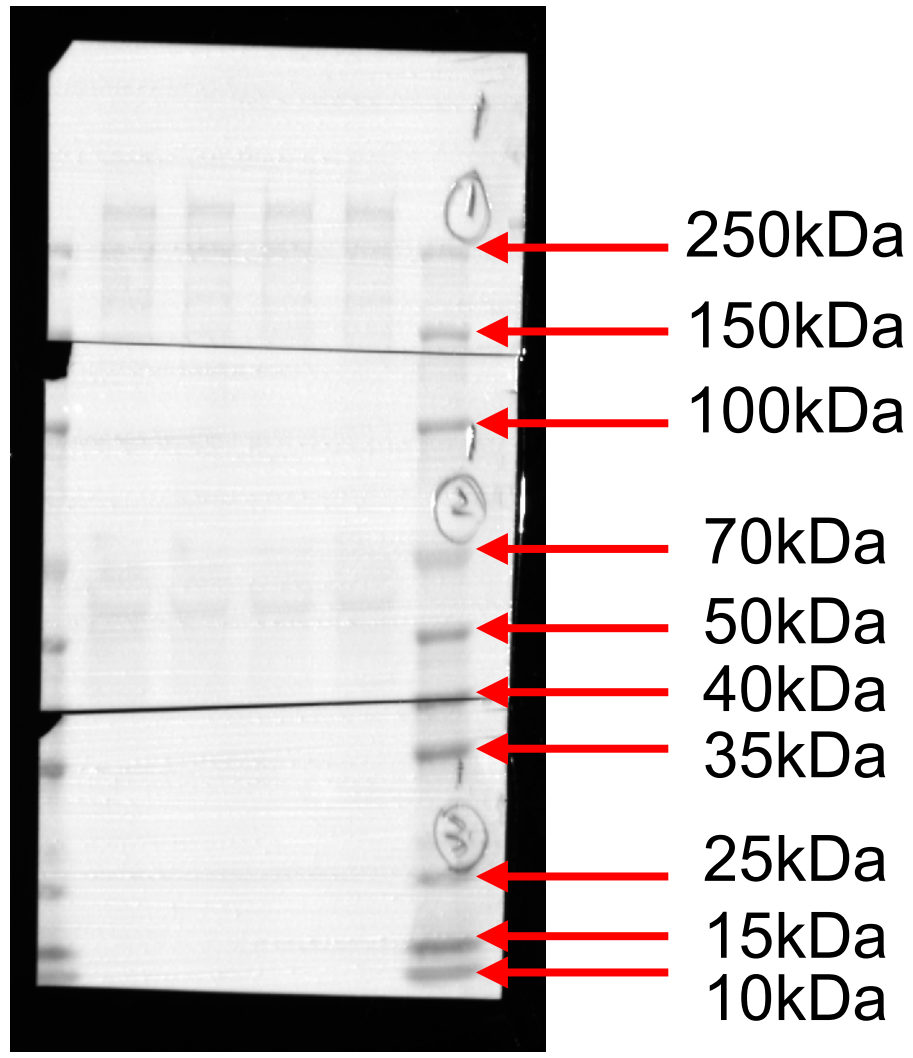

Full unedited gel for Figure 5D

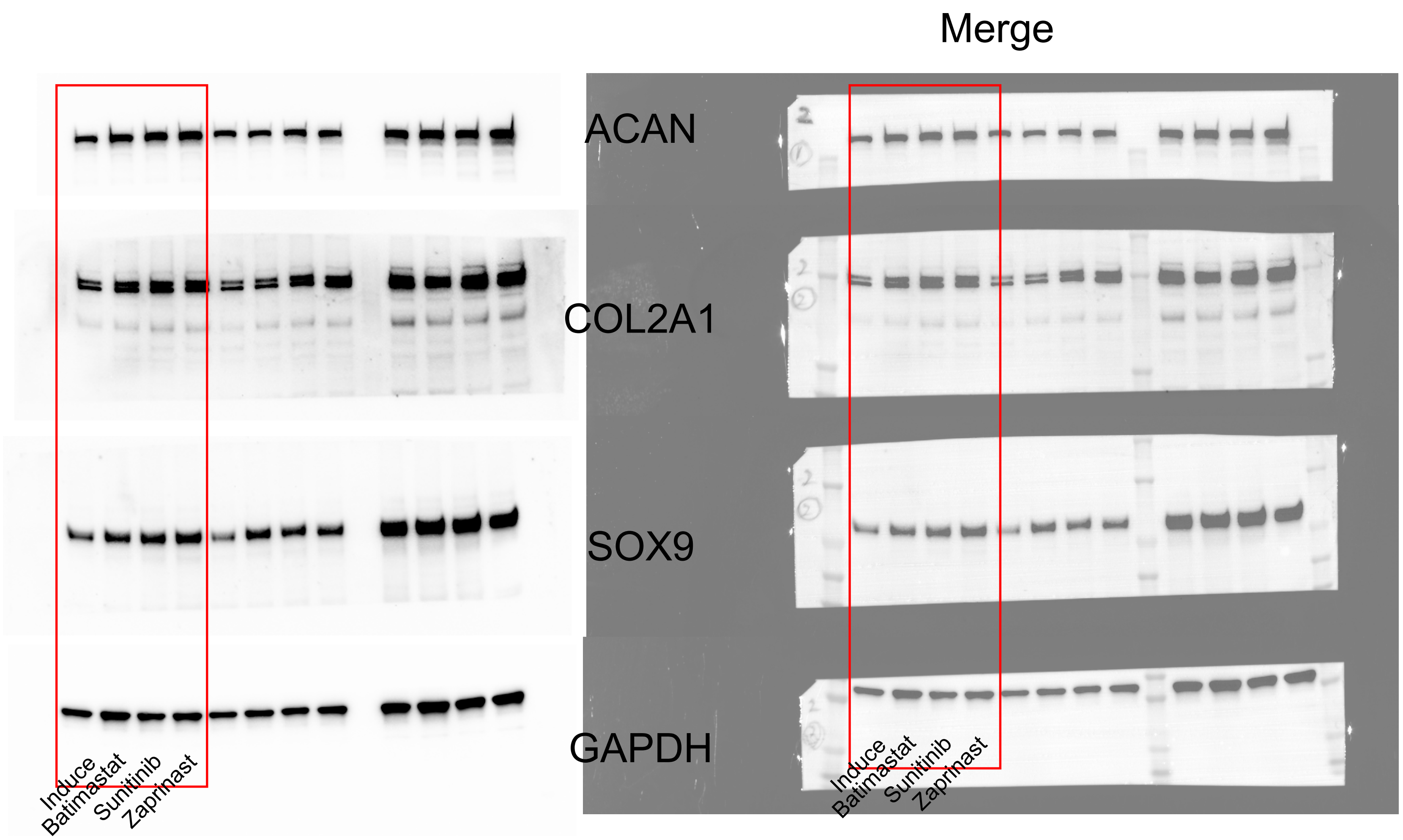

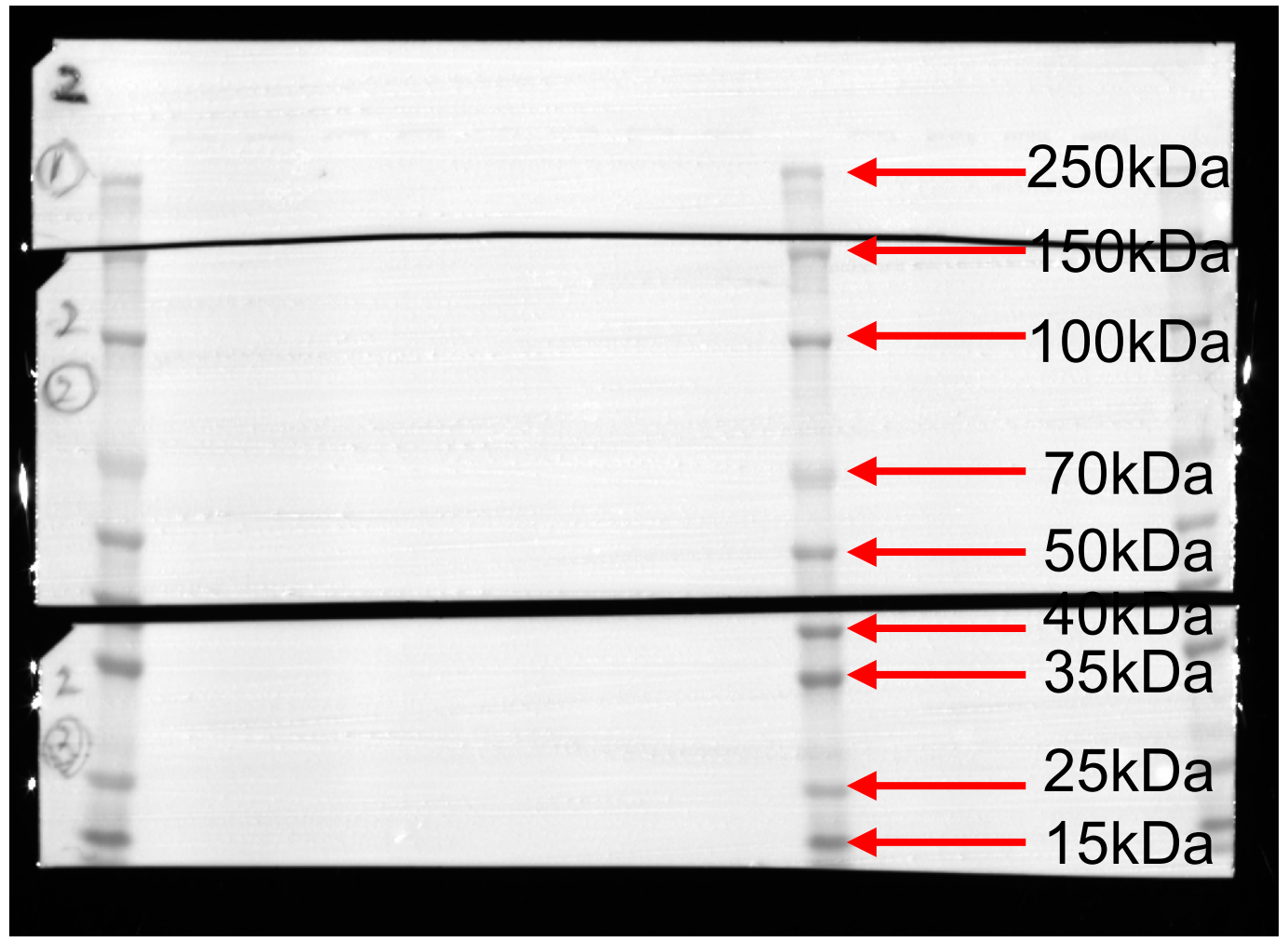

Full unedited gel for Figure 5N

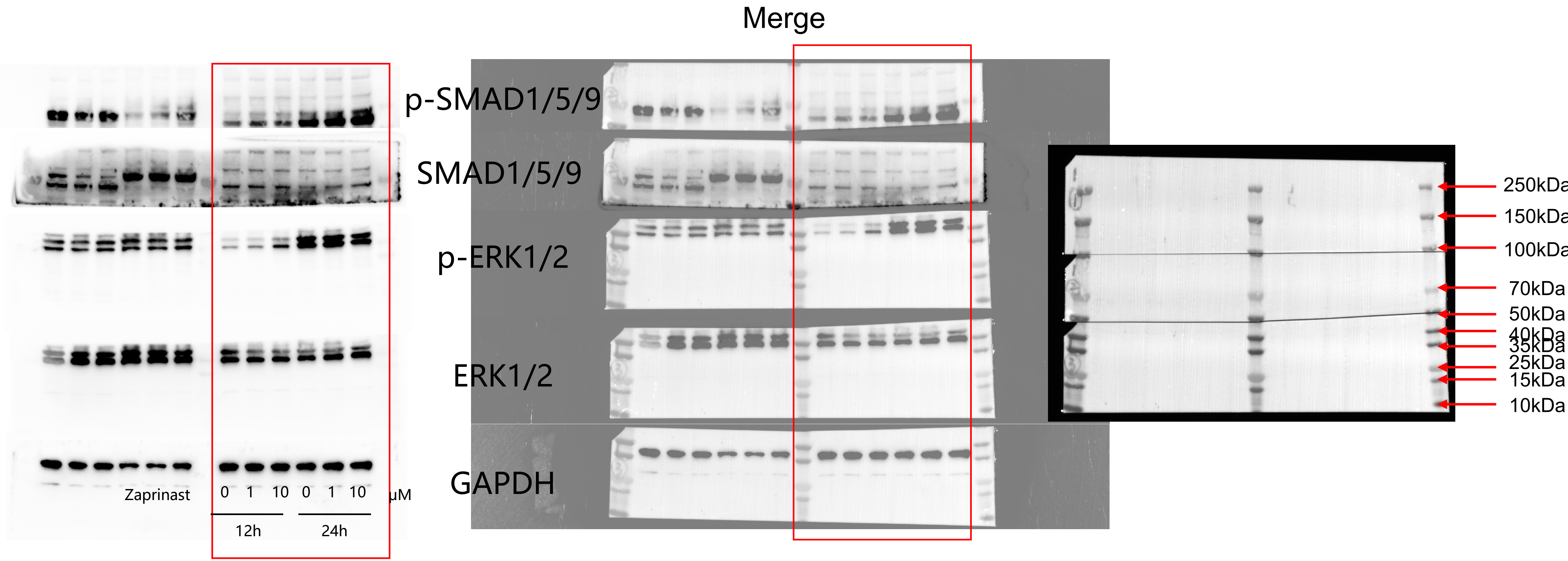

Full unedited gel for Supplemental Figure 13C

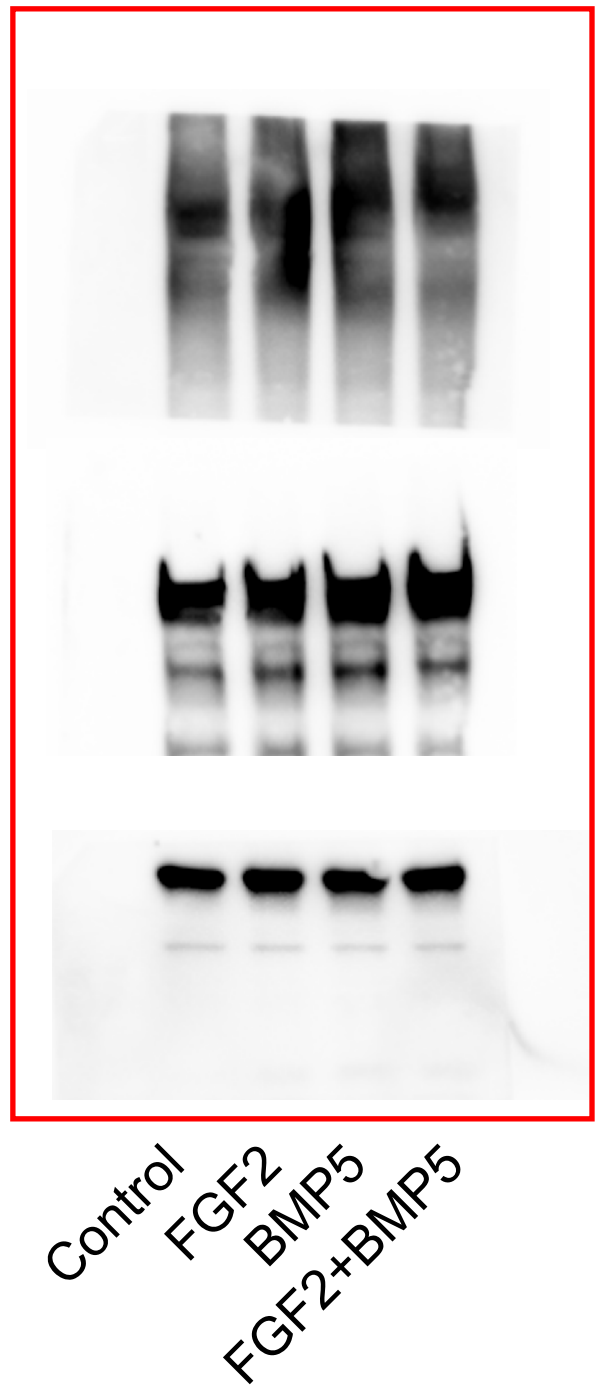

Merge

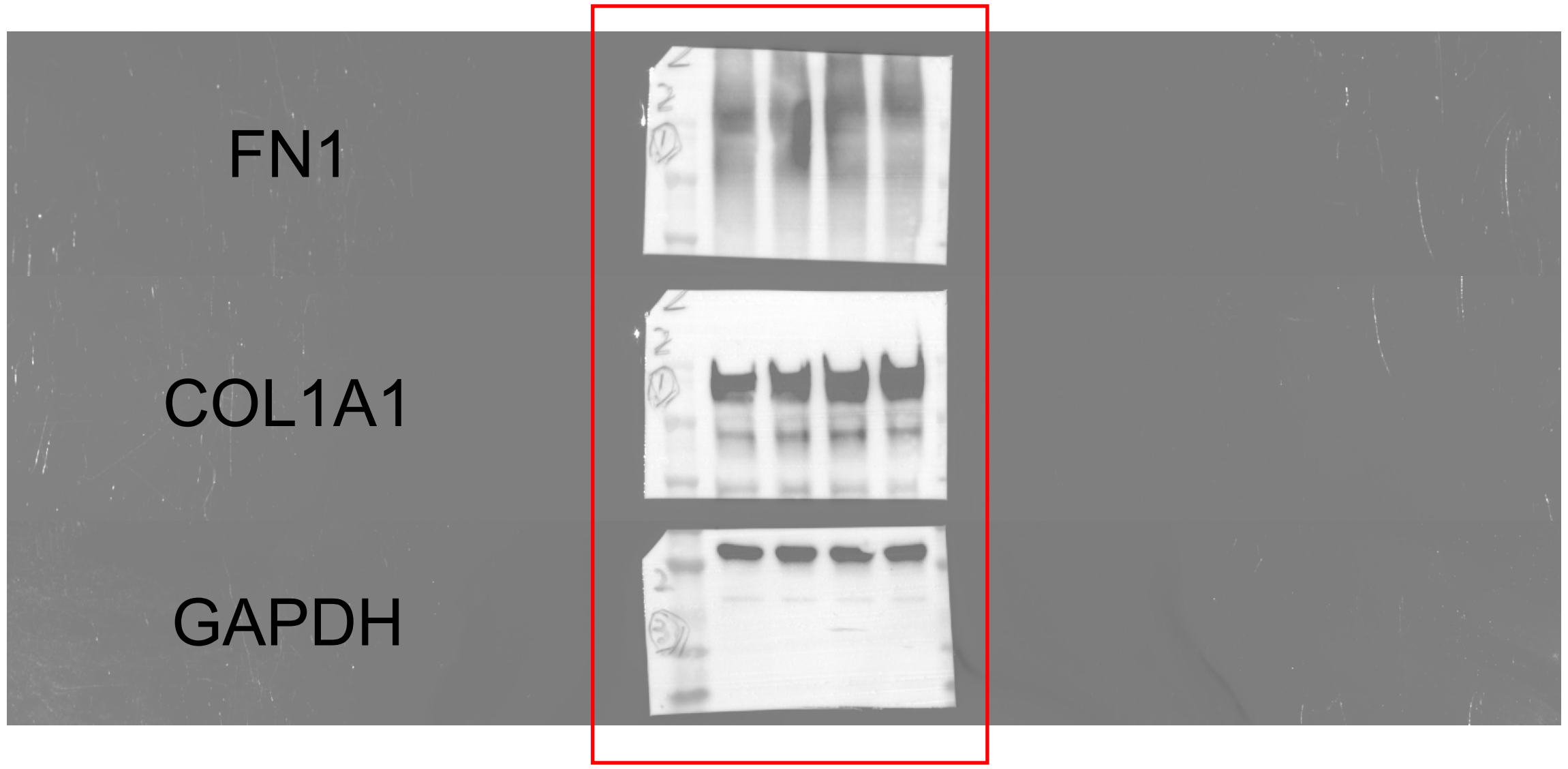

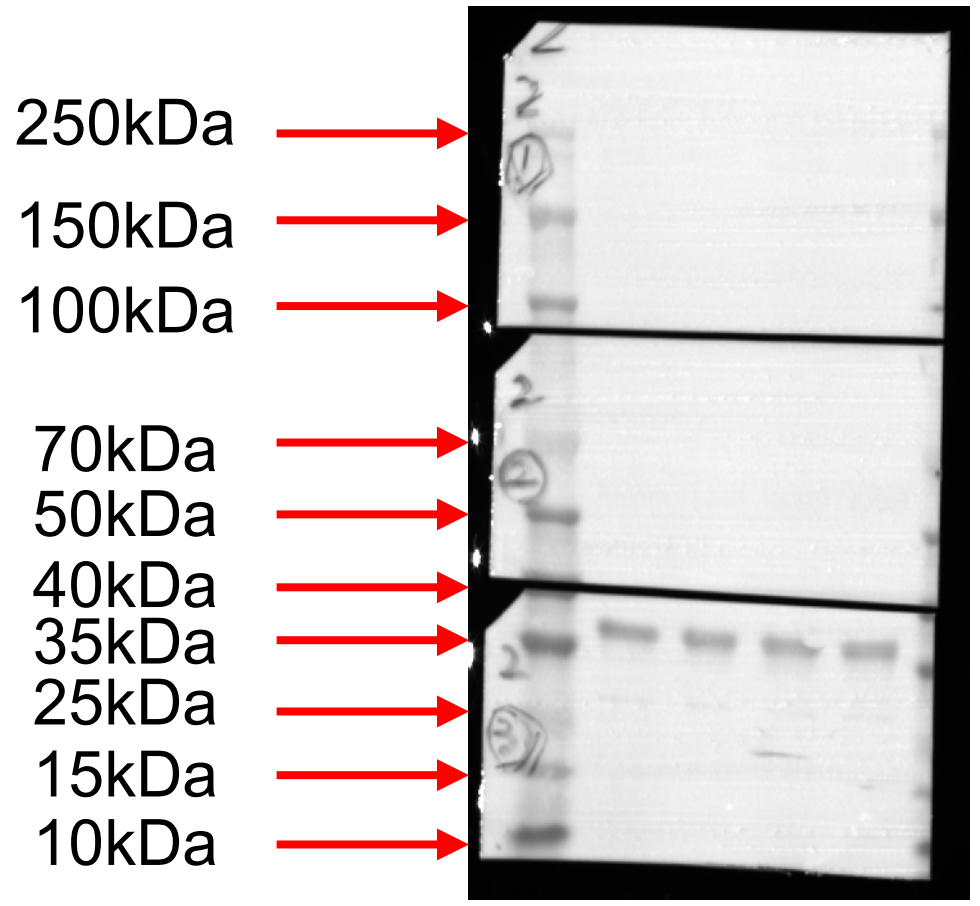

Full unedited gel for Supplemental Figure 13D

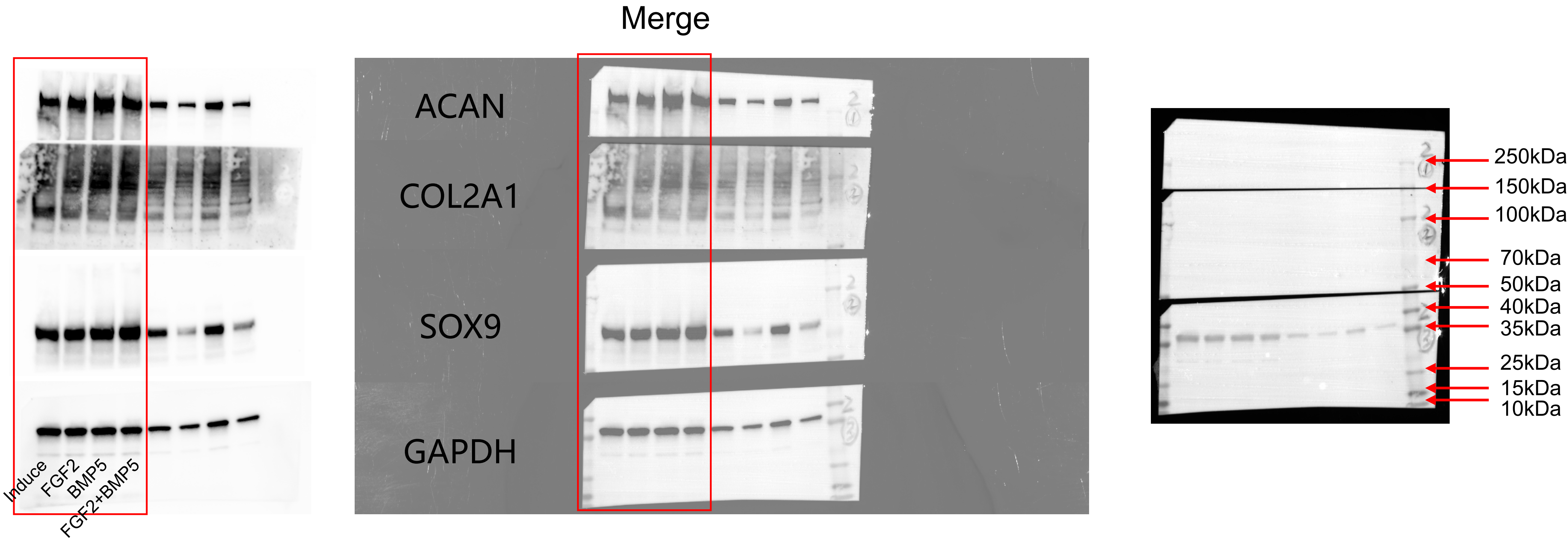

Supplement: Supplementary file 2 [file jciinsight-11-196343-s104.pdf]
